# Recurrent transitions to Little Ice Age-like climatic regimes over the Holocene

**DOI:** 10.1007/s00382-021-05669-0

**Published:** 2021-02-06

**Authors:** Samuli Helama, Markus Stoffel, Richard J. Hall, Phil D. Jones, Laura Arppe, Vladimir V. Matskovsky, Mauri Timonen, Pekka Nöjd, Kari Mielikäinen, Markku Oinonen

**Affiliations:** 1 Natural Resources Institute Finland, Ounasjoentie 6, 96200 Rovaniemi, Finland; 2 Climate Change Impacts and Risks in the Anthropocene (C-CIA), Institute for Environmental Sciences, University of Geneva, Geneva, Switzerland; 3 dendrolab.Ch, Department of Earth Sciences, University of Geneva, Geneva, Switzerland; 4 Department F.-A. Forel for Environmental and Aquatic Sciences, University of Geneva, Geneva, Switzerland; 5 School of Geography and Lincoln Centre for Water and Planetary Health, University of Lincoln, Lincoln, UK; 6 Climatic Research Unit, School of Environmental Sciences, University of East Anglia, Norwich, NR4 7TJ UK; 7 Laboratory of Chronology, Finnish Museum of Natural History, University of Helsinki, Gustaf Hällströmin Katu 2, 00014 Helsinki, Finland; 8 Institute of Geography RAS, Staromonetniy pereulok 29, 119017 Moscow, Russia; 9 Natural Resources Institute Finland, Tietotie 2, 02150 Espoo, Finland

**Keywords:** Little Ice Age, East Atlantic pattern, Atlantic meridional overturning circulation, Volcanic forcing, Paleoclimatology, Tree ring

## Abstract

Holocene climate variability is punctuated by episodic climatic events such as the Little Ice Age (LIA) predating the industrial-era warming. Their dating and forcing mechanisms have however remained controversial. Even more crucially, it is uncertain whether earlier events represent climatic regimes similar to the LIA. Here we produce and analyse a new 7500-year long palaeoclimate record tailored to detect LIA-like climatic regimes from northern European tree-ring data. In addition to the actual LIA, we identify LIA-like ca. 100–800 year periods with cold temperatures combined with clear sky conditions from 540 CE, 1670 BCE, 3240 BCE and 5450 BCE onwards, these LIA-like regimes covering 20% of the study period. Consistent with climate modelling, the LIA-like regimes originate from a coupled atmosphere–ocean–sea ice North Atlantic-Arctic system and were amplified by volcanic activity (multiple eruptions closely spaced in time), tree-ring evidence pointing to similarly enhanced LIA-like regimes starting after the eruptions recorded in 1627 BCE, 536/540 CE and 1809/1815 CE. Conversely, the ongoing decline in Arctic sea-ice extent is mirrored in our data which shows reversal of the LIA-like conditions since the late nineteenth century, our record also correlating highly with the instrumentally recorded Northern Hemisphere and global temperatures over the same period. Our results bridge the gaps between low- and high-resolution, precisely dated proxies and demonstrate the efficacy of slow and fast components of the climate system to generate LIA-like climate regimes.

**Supplementary Information:**

The online version contains supplementary material available at 10.1007/s00382-021-05669-0.

## Introduction

Major climate episodes have interrupted the ongoing interglacial period (Denton and Karlén 1973; Bond et al. 2001; Mayewski et al. 2004; Wanner et al. 2011). The latest of these episodes, the ‘Little Ice Age’ (LIA), has excited much interest with extensive evidence from a broad range of early instrumental and documentary sources and environmental proxies (Lamb 1995; Grove 2004). Many records from around the North Atlantic and European domains demonstrate a marked cold period between 1570 and 1900 CE (Matthews and Briffa 2005) and those across the Arctic region between 1600 and 1850 (Kaufman et al. 2009). External forcing by solar minima (Eddy 1977a, b) and explosive volcanism (Bradley and Jones 1993) has for long been suggested as multiple drivers (Owens et al. 2017) of these variations, with their induced changes in the ocean-atmospheric circulation in the northern Atlantic being presumed to explain the prevalence of cool climates (Lamb 1979). Recently, evidence of sudden anomalous volcanic forcing (Sigl et al. 2015; Stoffel et al. 2015; Toohey et al. 2019) driving a first millennium CE climatic downturn, potentially analogous to the LIA, has been presented and casts a new focus on LIA-like climatic regimes through temperature-dependent records illustrating prolonged cold climates. A regime of cold events has been described between 300 and 900 CE (Wanner et al. 2011; Helama et al. 2017a, b), this interval being further punctuated by an episode of abrupt, synchronous, multi-decadal (Larsen et al. 2008; Helama et al. 2017b) or even centennial (Büntgen et al. 2016; Matskovsky and Helama 2016) cold climate anomalies across a collection of Northern Hemisphere (NH) proxy sites around the mid-sixth century.

Despite the demonstration of anomalous climate and environmental conditions, however, many questions remain. Two competing hypotheses exist to account for the existence of LIA-like colder periods. First, these LIA-like periods are suggested to occur in a series of late Pleistocene and Holocene ice rafting episodes associated with a 1000–2000 year cooling rhythm in North Atlantic surface waters, in keeping with millennial solar minima and with the potential involvement of weaker Atlantic Meridional Overturning Circulation (AMOC) (Bond et al. 2001). Widespread palaeoclimatic evidence supports the coincidence of these oceanic swings with a cluster of proxy indications for cold events (e.g. Berglund 2003; Wanner et al. 2011; Helama et al. 2017a). Second, the palaeoceanographic hypothesis is challenged by well-dated proxy and modelling evidence demonstrating distinct cold temperature anomalies being triggered by volcanic forcing (Zhong et al. 2011; Miller et al. 2012; Slawinska and Robock 2018). Tree-ring proxies portray distinct cold temperature anomalies from 536 CE onwards, over the next two or three decades (Larsen et al. 2008; Helama et al. 2017b). While a temperature response to the volcanic forcing is found in model simulations between 536 and 545 CE (Toohey et al. 2016), it has been proposed that the cold climates may have been sustained until the seventh century CE, at least in a limited number of Eurasian proxy sites (Büntgen et al. 2016; Matskovsky and Helama 2016). The hypothesis of volcanic forcing triggering a prolonged, widespread centennial-scale cooling is largely built on climate modelling whereby sea-ice and oceanic feedback mechanisms reinforce the persistence of cold conditions to prevail much longer than the primary forcing by volcanic aerosols (Zhong et al. 2011; Miller et al. 2012). As such, internally driven sea-ice and oceanic factors, similar to the palaeoceanographic hypothesis, have been put forward to explain the LIA-like cold periods in both low- and high-resolution proxy records.

There is also the question of how the LIA-like events of the past 2000 years relate to similar centennial-scale climate downturns that occurred earlier in the Holocene, however, previous inferences have mainly been gathered from lower-resolution proxy records that were less well-dated (Lamb 1995; Bond et al. 2001; Mayewski et al. 2004; Wanner et al. 2011) than those records used to explore the LIA-like climate conditions over the Common Era. Dating of these BCE events has not yet been agreed upon (Table S1). Coarser temporal resolution and lower dating accuracy may play a role in this discrepancy. Another factor may relate to the definition of LIA-like conditions in proxy records that have been recently challenged by a dendroclimatic analysis suggesting that the climatic regime during the LIA could actually be interpreted in the context of multiple tree-ring records sensitive not only to temperature but also to irradiance and/or cloud cover variables and correspondingly linked with large-scale atmospheric patterns (Gagen et al. 2011; Young et al. 2012; Loader et al. 2013). This would suggest that analysing only temperature-sensitive records may be an inefficient approach to detect LIA-like climatic regimes. The value of integrating tree-ring reconstructions of summer temperature (based on physical tree-ring growth) and cloud cover (based on isotopic ratios) into a new summation record, has also been demonstrated to indicate the particular changes in large-scale circulation inherent to the climatic regime of the LIA over the North Atlantic-Arctic sector, but so far not applied beyond the past millennium (see Young et al. 2012; Loader et al. 2013). Here we aim to identify Holocene periods with cold and clear sky conditions from much longer tree-ring chronologies and, importantly, use such data to detect LIA-like climatic regimes over the mid and late Holocene times. Moreover, the reconstructed shifts in past climate variability are compared with the records indicative of the atmospheric and oceanic changes in the North Atlantic-Arctic sector over the same time, in addition to records of solar forcing and volcanic events. To do so, we produce and analyse a new 7500-year long palaeoclimate record tailored to detect LIA-like climatic regimes from northern European tree-ring data. In addition to tree-ring based temperature records (Helama et al. 2010; Matskovsky and Helama 2014), we make use of a 7510-year tree-ring δ^13^C dataset representing high-latitude sites of northern Europe (Fennoscandia) for which carbon isotope fractionation by trees can be attributed to factors driving their CO_2_ assimilation. The δ^13^C anomalies in these datasets thus represent variations in irradiance and/or cloud cover, and consequently have the potential to reconstruct high- to low-frequency variability in cloud cover (Young et al. 2012; Helama et al. 2018a), i.e. an essential parameter of climate change (Stephens 2005) whose past variations are still only poorly understood. Crucially, our tree-ring δ^13^C-based cloud cover (Helama et al. 2018a) and temperature records (Helama et al. 2010; Matskovsky and Helama 2014) span the past 7510 years and are integrated to produce a new palaeoclimate dataset that can be used as a diagnostic tool to infer past LIA-like climate period since 5500 BCE.

Our tree-ring sites are located downstream of the North Atlantic region and are proximal to the Arctic which suggests that an interaction of large-scale atmospheric associations and climatic mechanisms could be inferred from the data. In this respect, the tree-ring records are hypothesised to record climate variability evolving from a coupled system comprising the atmosphere–ocean–sea ice interactions in the North Atlantic-Arctic sector. This is essential to further our understanding of potentially prolonged LIA-like conditions that could have been sustained over wider spatial scales (Miller et al. 2012; Slawinska and Robock 2018). This necessitates understanding of atmospheric pressure anomalies involved in influencing sea-ice accumulation in the Arctic and leading to freshwater fluxes into the Atlantic (Ionita et al. 2016), with climatic implications of the AMOC weakening (Jackson et al. 2015). The leading mode of North Atlantic atmospheric variability, the North Atlantic Oscillation (NAO), describes most of the atmospheric pressure fluctuations over the North Atlantic and drives the climate variability over much of Europe on synoptic to multi-decadal time scales (Hurrell and Deser 2010). It can be described as a fluctuation of atmospheric mass between two nodes: semi-permanent high and low pressure regions over the Azores and Iceland respectively. The NAO also interacts with the Atlantic Multidecadal Oscillation (AMO) (Folland et al. 2009), a multidecadal-scale fluctuation of ocean-wide mean sea surface temperatures (SST) over the North Atlantic (Enfield et al. 2001), and thus potentially with the AMOC. However, in agreement with recent proxy and modelling studies (Moffa-Sánchez et al. 2014; Rao et al. 2017), it is hypothesised that an even wider spectrum of atmospheric indices is needed to describe patterns of atmospheric response driven by solar and volcanic forcing, which are instrumental in producing the LIA-like climatic regimes. The East Atlantic Pattern (EAP) and Scandinavian Pattern (SCA) (Barnston and Livezey 1987) are the second and third modes of North Atlantic atmospheric pressure variability. The positive phase of the EAP is characterised by a monopole of low pressure to the west of Ireland, while the positive SCA features high pressure over Scandinavia. Here we use these additional indices to interpret the observed cloud cover/temperature shifts following volcanic events (Kobashi et al. 2017; Sigl et al. 2015) and solar forcing (Steinhilber et al. 2009) over the past 7500 years.

Our analysis presents a critical, high-resolution assessment on the LIA-like climate periods and the forcing mechanisms leading to their Holocene recurrence.

## Material and methods

### Palaeoclimate and forcing data

**Fig. 1:**
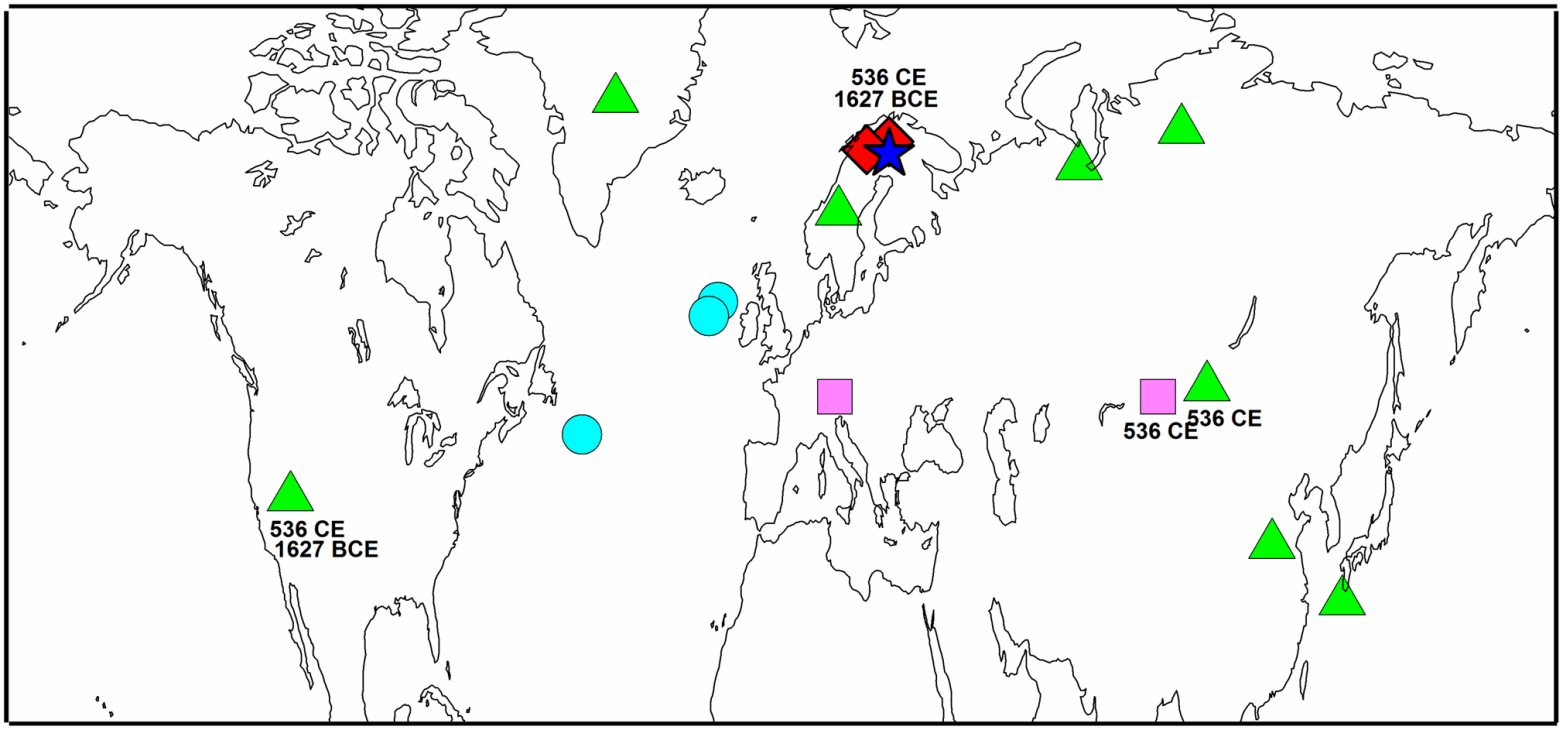
A map of showing the sites of palaeoclimatic proxy records. Our sites of maximum latewood density data for temperature reconstruction (Matskovsky and Helama 2014) are marked with diamond symbols and of tree-ring width and δ^13^C data for temperature and cloud cover reconstructions (Helama et al. 2010, 2018a) with star symbol. Also shown are the sites discussed in Sect. 4: the North Atlantic sites of North Atlantic ice-rafted debris (circles) (Bond et al. 2001), the tree-ring sites showing the evidence of cool temperatures confined to the ‘Late Antique Little Ice Age’ (536–660 CE) (rectangles) (Büntgen et al. 2016) and the other well-dated Northern Hemisphere proxy records illustrating the 536–570 CE climatic cooling (triangles) (Helama et al. 2017b). Frost ring evidence for years 536 CE and 1627 BCE are shown with respective calendar years from Finnish Lapland (Helama et al. 2019), western North America (Salzer and Hughes 2007), Mongolia (D'Arrigo et al. 2001) and the Altai mountains in central Asia (Churakova (Sidorova) et al. 2014)

The sites of palaeoclimate proxy data include northern Fennoscandia (Fig. 1) where several investigations have identified substantial amounts of pinewood preserved in subaerial conditions and as subfossils in the sedimentary archives of relatively small lakes (Eronen et al. 1999, 2002; Helama et al. 2015, 2019). Tree-ring samples of such subfossil remains and living Scots pine (*Pinus sylvestris* L.) trees have been previously utilised for reconstructions of summer temperature and cloud cover based on the stable carbon isotope values (δ^13^C) (Helama et al. 2018a), tree-ring width (TRW) (Helama et al. 2010) and maximum latewood density (MXD) chronologies (Matskovsky and Helama 2014). These reconstructions have been statistically calibrated and successfully verified against the instrumental data in their original publications, i.e. the TRW, MXD and δ^13^C data against the July mean temperature (Fig. S1), June–August mean temperature (Fig. S2) and June–August average cloud cover (Fig. S3), respectively (see Table S2 for online data availability). Decadal records were produced by averaging annually resolved reconstructions (Helama et al. 2010; Matskovsky and Helama 2014) for 10-year periods (2001–2010, 1991–2000, 1981–1990 CE…) for comparisons. Records of z-scores (original data normalised to a mean of zero and standard deviation of one) were produced over the periods common to tree-ring δ^13^C, TRW and MXD based cloud cover and temperature reconstructions. The MXD based temperatures were available from 1 CE (see Matskovsky and Helama 2014), whereas the reconstructions based on tree-ring δ^13^C and TRW were available from 5500 BCE (Helama et al. 2010, 2018a). The values of summation (*S MXD*_*t*_) curve based on δ^13^C and MXD records for year *t* (with positive and negative values for CE and BCE years, respectively) were calculated as

1 $$S MXD_{t} = \frac{{\left( {C_{t} - C_{x} } \right)}}{{C_{sd} }} + \frac{{\left( {T MXD_{t} - T MXD_{x} } \right)}}{{T MXD_{sd} }},\quad t \ge 1,$$

and the values of summation (*S TRW*_*t*_) curve based on δ^13^C and TRW records were calculated as

2 $$S TRW_{t} = \frac{{\left( {C_{t} - C_{x} } \right)}}{{C_{sd} }} + \frac{{\left( {T TRW_{t} - T TRW_{x} } \right)}}{{T TRW_{sd} }}, \quad t \ge - 5500,$$

where *C*_*t*_, *T MXD*_*t*_ and *T TRW*_*t*_ are the reconstructed values for cloud cover and temperature in year *t*, respectively, *C*_*x*_, *T MXD*_*x*_ and *T TRW*_*x*_ are the means of the respective values over their common period, and *C*_*sd*_, *T MXD*_*sd*_ and *T MXD*_*sd*_ are the corresponding standard deviations calculated over the same period (in order to produce the z-scores of cloud cover and temperature data). These were calculated similar to those of Loader et al. (2013) who calculated the difference between their temperature and sunshine reconstructions. Since the sunshine variables are highly negatively correlated with the cloud cover over the summer season, the summation curve produced here shows negative values of the curve for periods of cold and sunny, positive values warm and cloudy conditions, and in that way in a fashion similar to Loader et al. (2013).

Confidence limits were defined for the summation curve by bootstrapping. Randomly selected blocks (with lengths determined similar to Adams et al. 2003) of z-score values of reconstructed temperatures and cloud cover were summed one thousand (1000) times and the confidence limits were approximated by the corresponding percentiles of the resulting sampling distribution of that statistic. The time-series of total solar irradiance reconstruction (Steinhilber et al. 2009) and volcanic impact index (Kobashi et al. 2017), available on 5-year and 1-year resolutions, respectively, were averaged for 10-year periods to correspond to our tree-ring based reconstructions. All these data were further averaged for 70-year periods to compare them with the palaeoceanographic records from the North Atlantic (Bond et al. 2001) (Fig. 1) available at this lower resolution. These records are based on petrological tracers in the form of hematite-stained grains, volcanic glass from Iceland and detrital carbonate percentages of lithic grains (ice-rafted debris; IRD) in a sedimentary particle size-range of 63–150 microns from deep-sea sediment cores. Palaeooceanographic records were contrasted both visually and statistically with the abovementioned climate and forcing proxy records.

### Statistical analyses

The different records were compared visually and by using Pearson correlations. Statistical significance for the resulting Pearson correlations were assessed using 100,000 Monte Carlo simulations using surrogate data produced in accordance with the frequency–domain method, adopting published algorithms (Macias-Fauria et al. 2012). Climate anomalies characteristic of the post-eruption sequence were assessed using superposed epoch analysis (SEA), which is commonly applied when the possible responses of the climate system to volcanic forcing are investigated (Adams. et al. 2003; Jones et al. 2003; Sigl et al. 2015; Wilson et al. 2016; Helama et al. 2018b). Temperature, cloud cover and summation data were centred and averaged for the five and ten largest volcanic eruptions of the past 2000 years (Sigl et al. 2015) (Table S3), the centred decade including the year *t*. Bootstrapping was used to approximate the confidence limits for the SEA from surrogate palaeoclimate records produced by randomly selecting blocks (with lengths determined similar to Adams et al. 2003) of corresponding proxy-based values and calculating the epochal mean repeatedly one thousand (1000) times for each SEA to define the appropriate confidence limits from the resulting sampling distribution of the statistic. Alternative SEAs were reproduced for the normalised data which tended to remove any disproportionate weight any extreme case could have on the estimates (Adams et al. 2003). Here, the mean of each key date window was removed and the resulting values were divided by the minimum absolute value in that window. This process was expected to scale the magnitudes in each row of the eruption matrix so that any single anomaly should not excessively influence the resulting SEA pattern (Fig. S4).

### Instrumental climate data

Instrumentally recorded NH and global temperature data from the Climatic Research Unit (CRUTEM4) (Jones et al. 2012) was adopted over the 1851–2010 CE period for annual (January through December) and summer (June through August) seasons, smoothed with a 15-year spline function and correlated with similarly smoothed tree-ring proxy data over the same period. A set of EOF-based (empirical orthogonal function) circulation indices was derived from the first three principal components of the sea level pressure (SLP) data (Compo et al. 2011) (Twentieth Century Reanalysis) taken over − 90 to 40° W, 20–80° N for the 1880–2014 CE period. For summer these circulation indices resemble the NAO, SCA and EAP. Teleconnection indices of the North Atlantic Oscillation (NAO-CPC), East Atlantic Pattern (EAP-CPC) and Scandinavian Pattern (SCA-CPC), derived from 500 hPa GPH anomalies (Barnston and Livezey 1987) were smoothed with a 15-year spline function and correlated with similarly smoothed tree-ring proxy data over the 1951–2010 CE period. The NAO-index series available over extended period (1901–2010 CE) (Hurrell and Deser 2010), the index of the AMO, the raw, non-detrended AMO record (1861–2010 CE) (Enfield et al. 2001) and the EOF-SLP data (1881–2010 CE) were smoothed with a 15-year spline function and correlated with similarly smoothed tree-ring proxy data over their overlapping periods. All these instrumental datasets represent the summer season (June through August). These observational datasets were correlated with tree ring data over the instrumental period. See Table S2 for online availability of these data.

## Results

### Comparisons with instrumental data

**Fig. 2:**
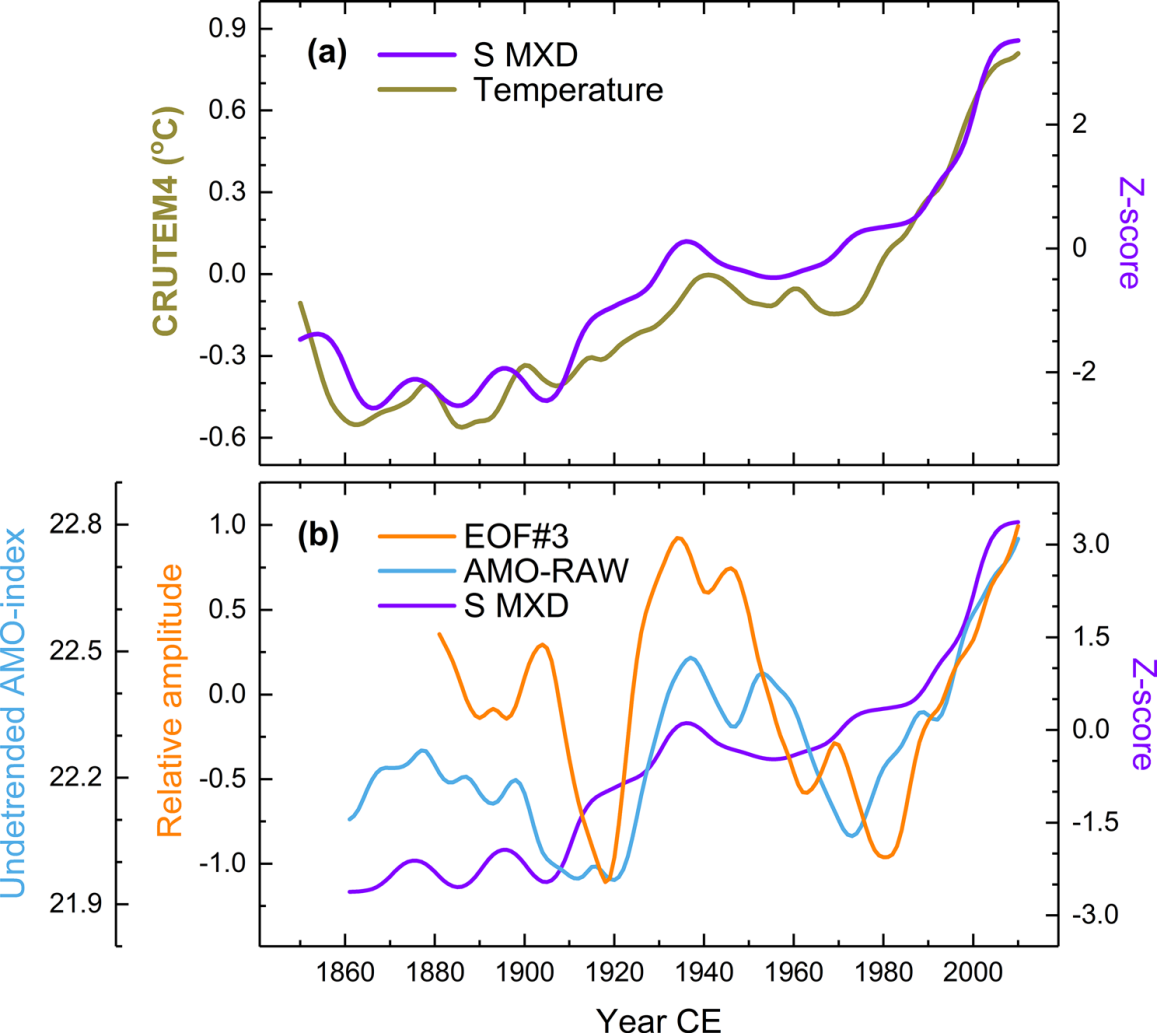
Global, oceanic and atmospheric associations in the proxy data. Comparisons between the summation curve (S) based on the maximum latewood density (MXD) and the global temperature record for annual season (CRUTEM4) over the 1851–2010 CE period (Jones et al. 2012) (**a**) and non-detrended AMO-record (AMO-RAW, 1861–2010 CE) (Enfield et al. 2001) and the third EOF based circulation index (EOF#3, 1881–2010 CE) (this study) (**b**). For correlations and their statistical significance, see Fig. 3

**Fig. 3:**
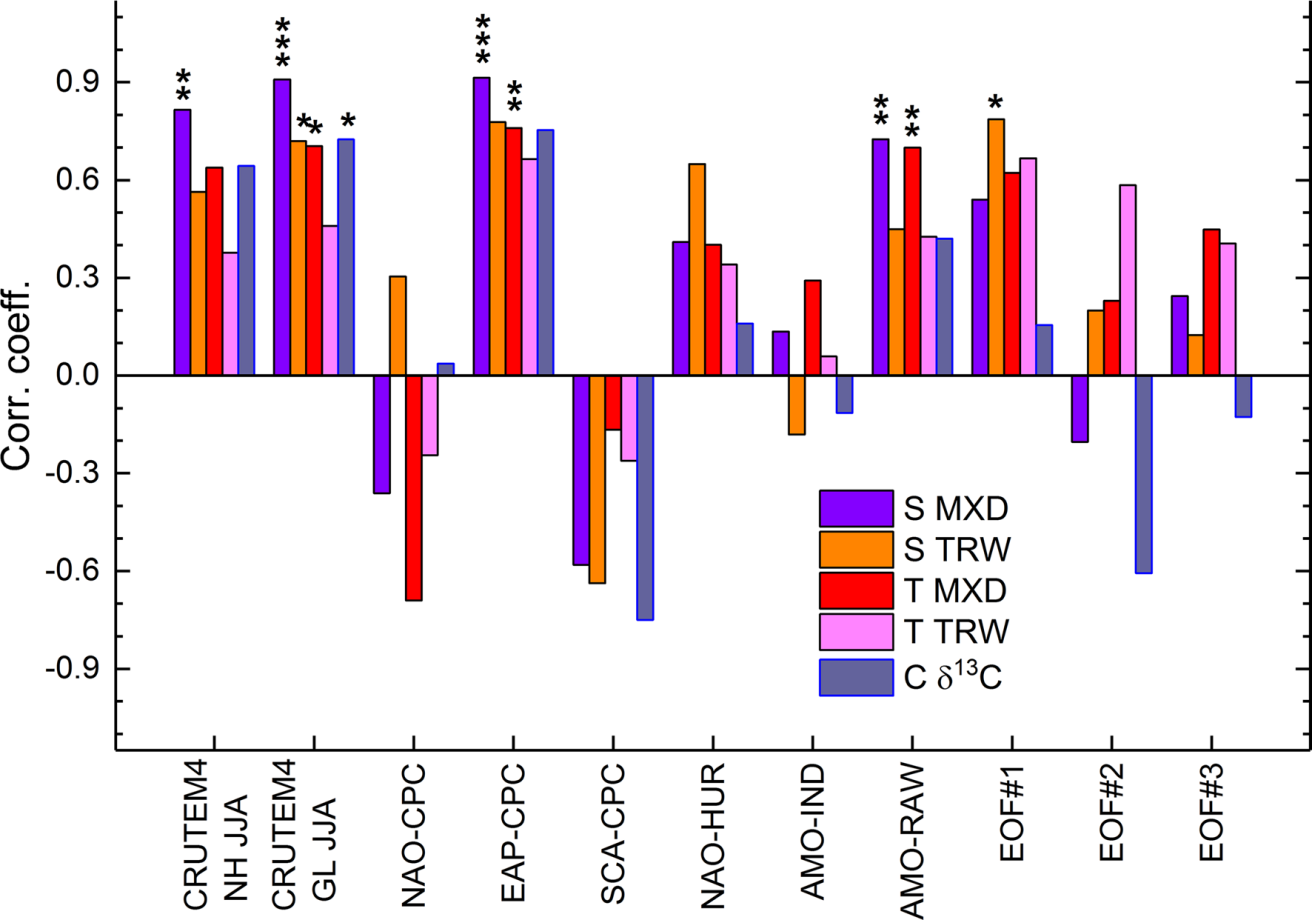
Pearson correlations of the proxy data to North Atlantic variability. Summation (S MXD (Eq. 1) and S TRW (Eq. 2)), temperature (T) and cloud cover (C) records based on the maximum latewood density (MXD), tree-ring width (TRW) and stable carbon isotope (δ^13^C) chronologies correlated with the Northern Hemisphere (NH) and global (GL) temperature records for summer season (JJA) over the 1851–2010 CE period, with the teleconnection indices of the North Atlantic Oscillation (NAO-CPC), East Atlantic Pattern (EAP-CPC) and Scandinavian Pattern (SCA-CPC) over the 1951–2010 CE (Barnston and Livezey 1987) and 1901–2010 CE (NAO-HUR) (Hurrell and Deser 2010) periods, with the index of the Atlantic Multidecadal Oscillation (AMO-IND) and the raw, non-detrended AMO-record over the 1861–2010 CE (AMO-RAW) (Enfield et al. 2001) and the EOF based circulation indices (this study), for summer season, over the 1881–2010 CE periods. Statistical significance at levels p < 0.05, p < 0.01 and p < 0.001 are denoted by one (*), two (**) and three asterisks (***), respectively, assessed using one hundred thousand Monte Carlo simulations (Macias-Fauria et al. 2012)

Tree-ring proxy records are highly correlated to the instrumentally recorded NH and global temperatures (Fig. 2a). Over decadal and longer scales, correlations are highest between the summation curve of temperature and cloud cover records and global land temperatures (Jones et al. 2012), for which they reach r ~ 0.9, which is statistically significant (Fig. 3) (p-value evaluations for correlations here and hereafter are based on a Monte Carlo procedure, see Methods). Our proxy data reproduces the warming from the nineteenth century to the 1930s, thus reliably detecting the LIA ending both on an annual and a seasonal basis in these instrumental data, as well as the rising temperatures from the 1960s onwards (Fig. 2a). Overall, the summation data is more strongly associated with instrumental temperature data, than the separately analysed proxy records.

The proxy-based variations can be explained by atmospheric and oceanic indices (Fig. 3). There are notably strong, positive relationships between the MXD-based summation curve, and the EAP-CPC series (1951–2010 CE) with r = 0.891 (p < 0.001). An almost as strong correlation (r = 0.762) is found between the summation curve and the EOF3 of the summer SLP data, indicative of EAP variations over an extended period (1881–2010 CE), this EOF data being also positively and statistically significantly associated with the AMO record (Fig. S5). These associations are reinforced by strong positive correlations between the summation curve and the non-detrended AMO record (1861–2010 CE), with r = 0.725 (p = 0.0025) (see also Fig. 2b).

Temperature records correlate positively with the summer EAP-CPC series (1951–2010 CE) and the non-detrended AMO and temperature records (Fig. 3) with r = 0.699 (p = 0.0044). Cloud cover records are positively connected to the EAP-CPC series; they also correlate negatively with the summer SCA-CPC series, but these correlations are not statistically significant based on the Monte Carlo evaluation. Compared to the summation curve, the temperature and cloud cover records are, when analysed separately, more weakly related to the instrumental data.

### Reconstructions over the Common Era

**Fig. 4:**
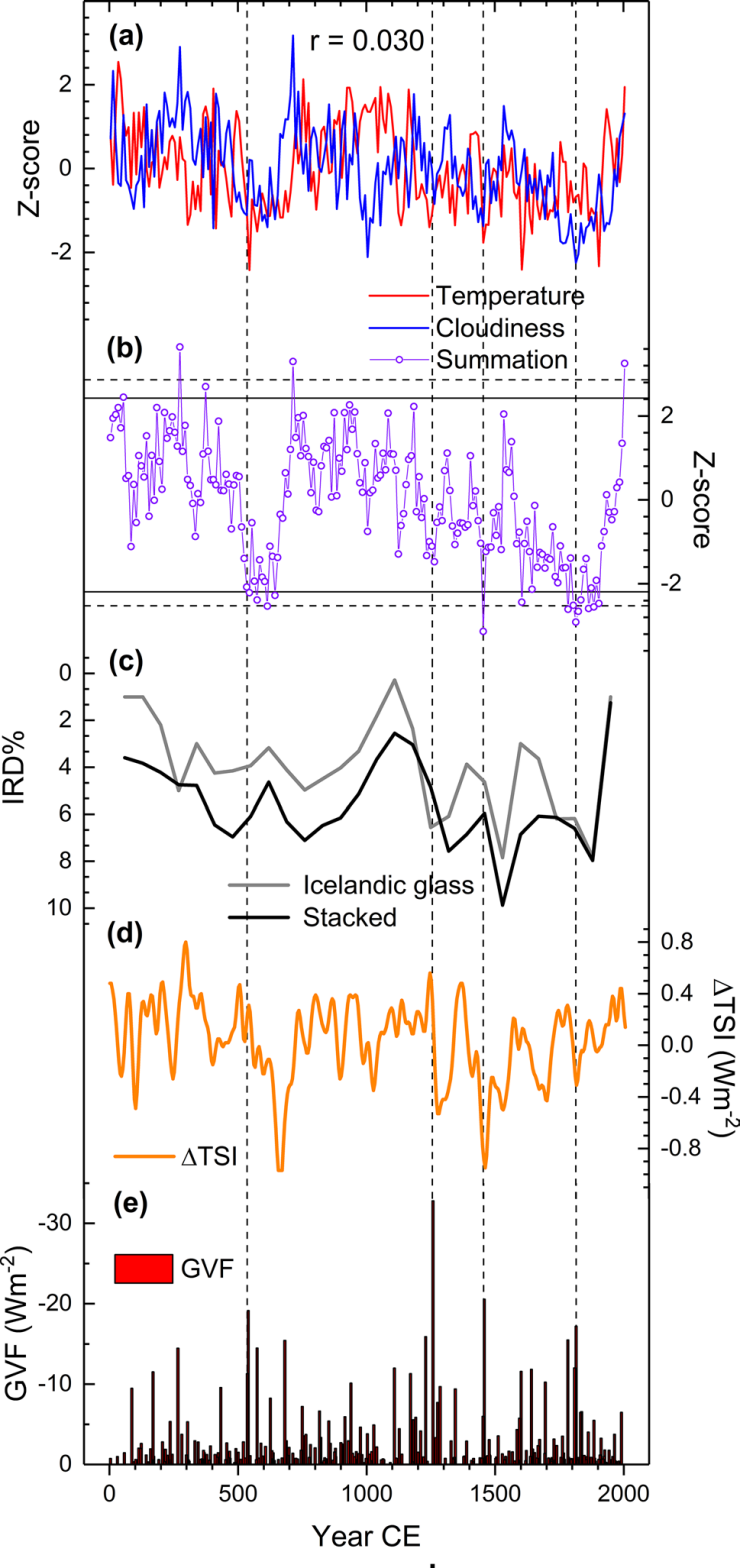
Common Era proxy records. Reconstructions of temperature and cloud cover transformed into z-scores (**a**) and the resulting summation curve (**b**) compared with records of North Atlantic ice-rafted debris (IRD%) (Bond et al. 2001) (**c**) and reconstructions of total solar irradiance (ΔTSI) (Steinhilber et al. 2009) (**d**) and global volcanic aerosol forcing (GVF) (Sigl et al. 2015) (**e**). The bootstrapped 2.5 and 97.5 percentiles (dashed horizontal lines) and 5 and 95 percentiles (continuous horizontal lines) are shown for the summation curve and the events in 536, 1257, 1450’s and 1809/1815 are indicated by vertical dashed lines

Temperature and cloud cover records show marked variations over the Common Era (Fig. 4a). Higher temperatures around 1000 CE are followed by notably colder conditions until the twentieth century. Cloudiness appears to vary around the temperature record, with notably low values recorded around 1800 CE. Although the temperature and cloud cover records do not generally correlate (r = 0.030, p = 0.7480), their resulting summation curve (Fig. 4b) does demonstrate progressively colder and less cloudy climates especially between 1600 and 1910 CE with extreme values between 1780 and 1910 CE. This trend is reversed with warmer and more overcast conditions over the past 100 years. Another period with an LIA-like climatic regime is found around the mid-first millennium CE, with strongest indications between 530 and 650 CE. Nonetheless, the two events also appear to differ in their structure. While the first millennium variation represents more of an abrupt and shorter (centennial) change in climate state, the change in second millennium conditions is a more gradual multi-centennial transition, with a cluster of low values during the nineteenth century CE.

Generally, the long-term climatic evolution can be seen to follow the IRD variations, with colder and less cloudy conditions during periods with higher IRD% (Fig. 4c), albeit the smoothness of the IRD record reduces the interpretive value of this connection. Solar minima in and around the 660's CE postdate the first millennium LIA-like event but that in the 1450’s CE is coeval to cold and less cloudy conditions during the 1450’s CE (Fig. 4d), which also coincides with volcanic forcing due to the 1450’s eruptions (Fig. 4e). It is notable that the post-eruption climatic events in 536/540 CE (Sigl et al. 2015) and 1809/1815 CE (Cole-Dai et al. 2009) were followed by centennial-scale regimes of LIA-like conditions. Further evidence for common forcing over a larger set of NH sites is obtained as frost rings are observed in 536 CE not only with our Finnish Lapland materials (Helama et al. 2019) but also in western North America (Salzer and Hughes 2007), Mongolia (D'Arrigo et al. 2001), the Altai mountains in central Asia (Churakova (Sidorova) et al. 2014) (see Fig. 1).

**Fig. 5:**
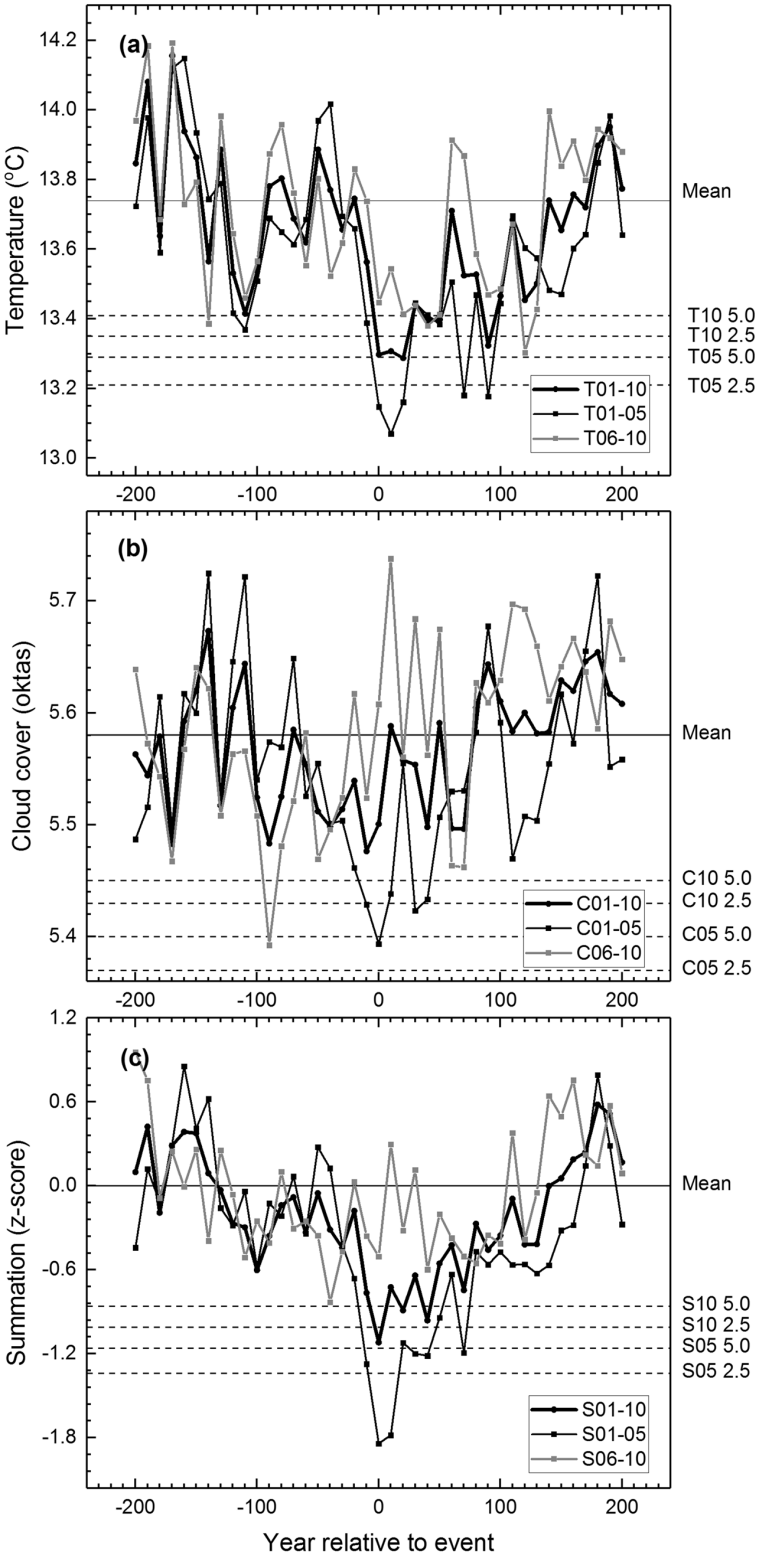
Superposed epoch analysis (SEA) for volcanic signatures. Temperature (**a**), cloud cover (**b**) and the summation data (**c**) centred on ten (T01-10, C01-10 and S01-10) and five (T01-05, C01-05 and S01-05) largest (and five next largest (T06-10, C06-10 and S06-10)) volcanic eruptions of the Common Era (Sigl et al. 2015) (see Table S3). Means are marked by continuous horizontal lines and the confidence limits by 2.5 and 5.0 percentiles (dashed horizontal lines)

This is also reflected in our superposed epoch analysis (SEA). The SEA responses for the largest eruptions (Table S3) demonstrate considerable multi-decadal to centennial drops in temperature (Fig. 5a) and to lesser degree, cloud cover (Fig. 5b) following the volcanic aerosol forcing, and similar evidence is found also for the summation data (Fig. 5c). Thus it appears that the volcanic signature we detect drives both the summertime cooling and reduced cloudiness as observed over a multitude of post-eruption decades. Noteworthy, the sixth to tenth largest eruptions are not followed by anomalous SEA responses (Fig. 5) suggesting that only the largest eruptions are responsible for this long-term signal. These results were not markedly altered when they were estimated for the normalised data (Fig. S6).

### Reconstructions since 5500 BCE

**Fig. 6:**
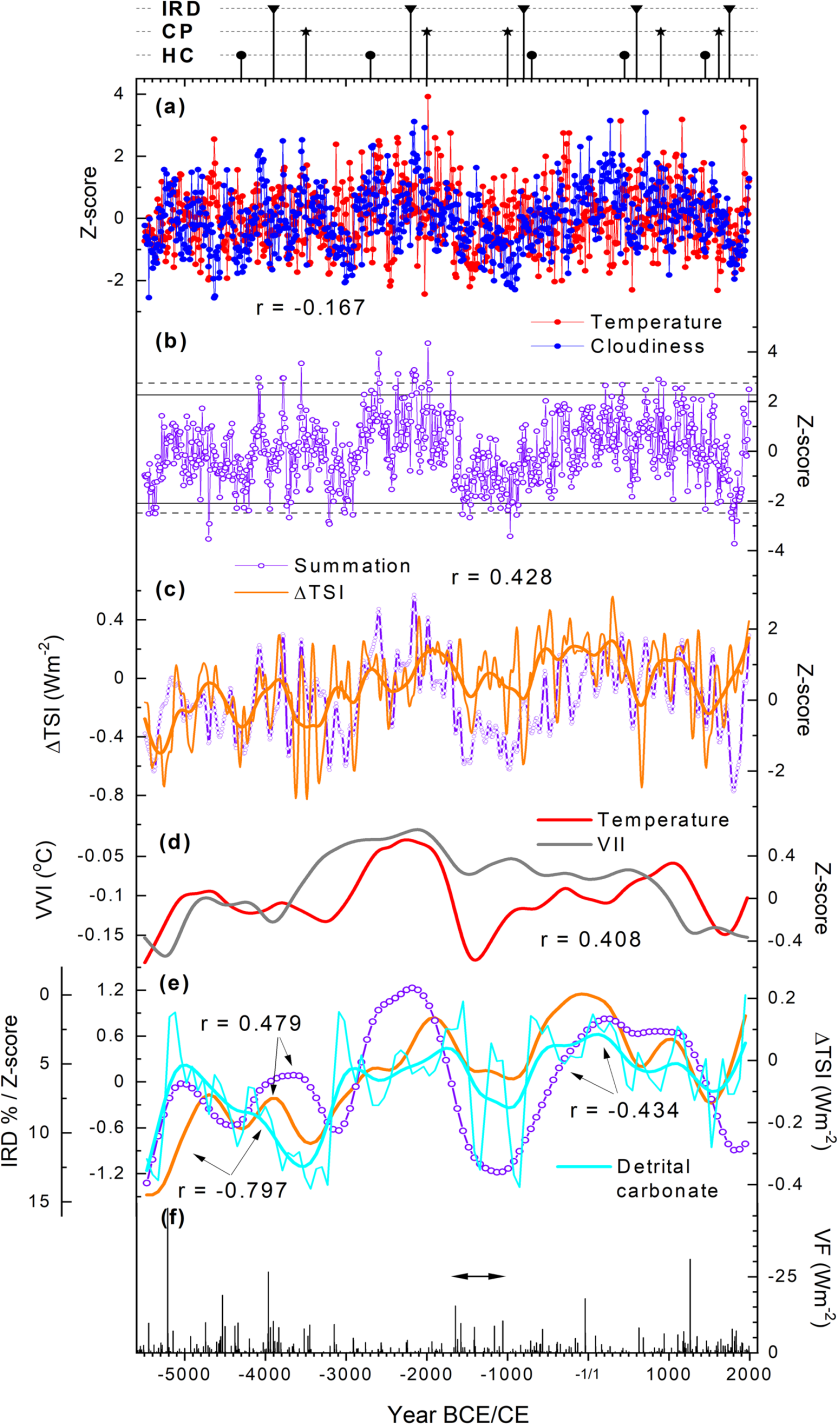
Proxy records of the past 7.5 thousand years. Reconstructions of temperature and cloud cover transformed into z-scores (**a**) and the resulting summation curve (**b**) compared with reconstruction of total solar irradiance (ΔTSI) (Steinhilber et al. 2009) (**c**), the volcanic impact index (VII) (Kobashi et al. 2017) (**d**) and with the records of North Atlantic ice-rafted debris (IRD%, detrital carbonate) (Bond et al. 2001) and ΔTSI (**e**) and volcanic forcing (VF) (Kobashi et al. 2017) (**f**). The bootstrapped 2.5 and 97.5 percentiles (dashed horizontal lines) and 5 and 95 percentiles (continuous horizontal lines) are shown for the summation curve (**b**). The records were filtered using the spline functions (thick lines) corresponding to 500-year (**c**) and 1000-year rigidities (**d**, **e**). An increased VF level from the mid second to early first millennium BCE is shown as horizontal arrow (**f**). Also shown (on the top), the timing of the North Atlantic ice-rafted debris (IRD) (Bond et al. 2001), the cool poles (CP) events (Mayewski et al. 2004) and the Holocene cold (HC) events (Wanner et al. 2011). For dates of the IRD, CP, and HC events, see Table S1

Temperature and cloud cover records exhibit a range of variations over the past 7510 years (Fig. 6a). They correlate with r = − 0.167 (p = 0.0070), but although the correlation appears statistically significant such a low coefficient means the result should be interpreted with caution. Positive anomalies indicative of warm and cloudy conditions are concentrated around the interval between 4.7 and 4.0 ka, this interval overlapping with the mid-Holocene warmth peaking between 5 and 4 ka as also indicated by various types of microfossil evidence in a wider region of northernmost Europe (Seppä et al. 2002; Helama et al. 2012). The two records show coincident events and, as a result, their summation curve shows, similar to the LIA extending to the nineteenth century CE, negative anomalies with at least five decadal summation estimates below the 5th percentile line observed here to start from 5450 BCE, 3240 BCE and 1670 BCE onwards (Fig. 6b). Each of these events is characterised by at least century-long, simultaneous drops in temperature and cloud cover and could be potentially linked with the ‘7.4 ka event’ (Filippidi et al. 2016), ‘5.2 ka event’ (Magny et al. 2006; Roland et al. 2015) and the sixteenth and seventeenth century BCE eruptive events (Salzer and Hughes 2007; Helama et al. 2019).

The temperature, cloud cover and the summation data correlate positively with the reconstruction of total solar irradiance (TSI; Steinhilber et al. 2009) with r = 0.091 (p = 0.0775), r = 0.109 (p = 0.0928), and r = 0.155 (p = 0.0114), respectively. These coefficients are, however, very low and should be interpreted cautiously. The summation curve appears most sensitive to solar forcing. Stronger indications are obtained on variations of longer term with r = 0.404 (p = 0.0087), r = 0.337 (p = 0.0266) and r = 0.428 (p = 0.0018), respectively, for temperature, cloud cover and their summation data with the TSI reconstruction (Fig. 6c). Correlating the records with leads/lags did not notably improve these relationships (Fig. S7). Also the North Atlantic data of ice-rafted debris (IRD) (Bond et al. 2001) compare more favourably with the summation curve rather than temperature or cloud cover records whereas the volcanic data correlate significantly only with our temperature record (see Fig. 6d, e, Fig. S8). While the correlations between the North Atlantic and solar forcing data are rather high (r ~ − 0.8) and statistically significant, those between the palaeoclimate and the forcing data are weaker with |r|~ 0.4 with both the IRD records and solar forcing data.

## Discussion

Solar and volcanic forcing have contributed to episodes of colder summer temperatures and reduced cloudiness that punctuate the Holocene climate history. Our findings are consistent with previous proxy analyses over the past millennium showing post-volcanic cooling and drier summer conditions largely in northern/north-west Europe over a number of years (Briffa et al. 1998; Stoffel et al. 2015; Rao et al. 2017). In contrast, Rao et al. (2017) suggests post-volcanic wetting over northern Europe, despite the hydroclimatic signal being recovered from the inverse association of high (low) summer temperatures with clear (overcast) skies in their underlying dataset (Cook et al. 2015). Here, the use of multiple proxy data allows a disentangling of the cloud cover and temperature signals from δ^13^C and MXD/TRW dataset. In addition to the multi-annual indications (Sigl et al. 2015; Rao et al. 2017), our data showed that the largest eruptions were followed by exceptional conditions which, in the case of temperature-related changes, persisted over several decades (the most sizeable anomalies were persisting over 20–30 years), and suggestively even over longer scales (Fig. 5). This response highlights the combination of climate conditions typical to the LIA-like climatic regimes in the region (Gagen et al. 2011; Young et al. 2012; Loader et al. 2013). This pattern of response also resembles the process of volcanically triggered LIA-like and internally sustained anomalies in climate models, whereby the post-volcanic LIA-like cool phase is maintained through ocean and sea-ice feedbacks with reduced poleward oceanic heat transfer, weakening of the AMOC, and even centennial-scale expansion of the NH sea ice extent (Miller et al. 2012; Slawinska and Robock 2018). Moreover, a recent modelling study emphasised the role of prolonged solar forcing fluctuations in amplifying the volcanically triggered sea-ice enhancement on decadal and centennial scales (Slawinska and Robock 2018). Such indications would resonate with the suggestions of a solar forcing influence on the IRD episodes and transmitted events in Earth’s climate system (Bond et al. 2001). Indeed, unlike the volcanic forcing (Kobashi et al. 2017), the solar forcing data (Steinhilber et al. 2009) shows strong correlations with the IRD records (Fig. S8), reaching considerably more negative coefficients here than those previously illustrated between the IRD records and solar forcing estimates (Bond et al. 2001). This illustrates how climate modelling may provide mechanisms to explain the tendencies in our tree-ring records towards colder and less cloudy conditions when the IRD% increased.

Further to these low-frequency connections, the climatic events following the eruptions most likely in 536/540, 1257 and 1809/1815 CE have been instrumental in initiating the anomalously cold centennial-scale NH conditions (Miller et al. 2012; Büntgen et al. 2016; Matskovsky and Helama 2016; Slawinska and Robock 2018). Moreover, the eruptions in the 1820's and 1830's CE have likely contributed to the sustained LIA conditions (Brönnimann et al. 2019). Here we note the role of cloud cover anomalies, coeval to low temperatures, in producing the LIA-like conditions during these events, which may help to detail the relevance of the potentially multiple forcing factors. It is notable that the summation curve shows markedly low values already for one to three decades prior to the foregoing events, thus, implying that coexisting influences from additional, non-volcanic factors may have paved the way for these events (Fig. 4b). In fact, the variations in the ocean-atmospheric circulation in the northern Atlantic have for long been seen as an explanation for the prevalence of cool climates (Lamb 1979). Negative departures in our proxy data could most likely be produced by decadal variations in the EAP or NAO phase, as determined from the correlations of proxy records and instrumental data (Fig. 3). While the negative summer NAO is expected to result in increased cloud cover in the region of our tree-ring sites (Folland et al. 2009), the negative summer EAP could indeed lead to less cloudy/less rainy conditions (Fig. S9) in accordance with the observed signal. Moreover, the positive correlations we found between the summation curve and the AMO record (Fig. 3) and between the EAP and AMO indices (Fig. S5) could suggest a shift toward the negative phase of the AMO to have occurred before and during the eruption dates. Continuation of the negative AMO phase as triggered by the nineteenth century CE eruptions has been suggested (Brönnimann et al. 2019). The cold (negative) AMO phase may also be indicated by the coincidence of increasing IRD% prior to these particular eruptions, indicating fluxes of colder surface waters in the Atlantic advected from Arctic sources, eventually leading to AMOC weakening (Bond et al. 2001).

Both the low solar irradiance and increased volcanic forcing have been shown to result in an atmospheric response that resembles a negative phase of the EAP (Moffa-Sánchez et al. 2014; Rao et al. 2017). These analogues demonstrate the likelihood of coexisting factors leading to the increasingly negative EAP-like phase during the periods representing the implied transitions towards LIA-like climatic regimes. The suggested atmospheric configuration is likely to promote a quasi-stationary high-pressure system off Western Europe and over the British Isles. The development of atmospheric blocking events strongly modifies the flow of westerlies, as shown during the solar minima of the past millennium (Moffa-Sánchez et al. 2014). As a result, the negative summer EAP phase leads to overall cooling over the continent and, contrary to the NAO (Folland et al. 2009), a meridional hydroclimatic gradient with decreased precipitation/cloudiness in the north (especially the British Isles, Fennoscandia and around the Baltic Sea) and increased precipitation in the south (Mediterranean) (Fig. S9). A consistent pattern of climatic response was also found showing dry conditions to prevail over northern Europe and wet conditions in the Mediterranean, in addition to overall continental cooling, following a modelled AMOC slowdown (Jackson et al. 2015). This hydroclimatic pattern should not be confused with the post-eruption effects of ‘dust veils’ that may have resulted in strong reductions in incoming solar radiation, as inferred from tree-ring δ^13^C records for a number of years after the past volcanic activity, the literature of such tree-ring signals including the 536/540 and 1815 CE events (Ogle et al. 2005; Helama et al. 2018b). The signals of these two types of anomalies (hydroclimatic and ‘dust veils’) are likely superimposed within the cloud cover record and at least the strongest ‘dust veil’ events may have attenuated the δ^13^C-based detection of hydrological response. This can be observed in our data at least for the mid-sixth century eruptions CE after which the long-term decline in cloud cover was disrupted by a phase of increased overcast conditions as recorded for the 540 s and 550 s CE (Fig. S10), in agreement with our previous analyses targeted on ‘dust veil’ signals in annually resolved δ^13^C data over the same period (Helama et al. 2018b).

The many internally driven and externally forced mechanisms may simultaneously act on different time scales, and correspondingly affect the observed spectrum of climate variability. This mirrors the proxy evidence for the ‘8.2 ka event’ (Bond et al. 2001; Mayewski et al. 2004; Wanner et al. 2011). Over this period, an abrupt climatic cooling event has been shown to punctuate a longer and less extreme period of colder and perturbed climate (Alley and Agústdottir 2005; Rohling and Pälike 2005), suggesting that the palaeoclimatic archive may actually contain anomalies on different time scales during such major events of climate cooling. Our tree-ring records do not extend over this early Holocene event, but indications suggest that cross-scale temporal interactions may be needed for a more complete explanation of the late Holocene LIA-like phenomena, consistent with recent climate modelling (Slawinska and Robock 2018). We note that increasing IRD%s are generally observed over the LIA, these values thus providing this period with even stronger implications of feedback mechanisms to account for its long-term climatic evolution. Similar to the LIA, the climatic downturn of the first millennium CE, widely discussed in palaeoclimate literature under the term ‘Dark Ages Cold Period’ (DACP) and comprising a range of proxy-indications of cold and disturbed climates between 400 and 765 CE (Helama et al. 2017a), has also been connected to the coinciding IRD event (Berglund 2003; Cui and Chang 2013; Li et al. 2016; Oliva and Gómez-Ortiz 2012; Reimann et al. 2011; Rudaya et al. 2016; Ruiz-Fernández et al. 2016; Ülgen et al. 2012; Zhong et al. 2014) (Fig. 4c; Table S1). Moreover, the DACP overlaps with those recorded as a Holocene cold event (300–500 CE) (Wanner et al. 2011) and between 450 and 700 CE across the Arctic (Kaufman et al. 2009). A more restricted use of ‘Late Antique Little Ice Age’ (LALIA) was recently proposed for isolating the post-536 CE cooling lasting to around 660 CE (Büntgen et al. 2016). However, such a precisely dated cooling (536–660 CE) may so far be confined to only a limited number of Eurasian sites (see Fig. 1) (Helama et al. 2017b), which include our data source (Matskovsky and Helama 2016), whereas the more rigorously documented cooling of shorter term (536 to no longer than 570 s CE) have been commonly presented in the context of the 536/540 CE ‘double event’ (Toohey et al. 2016), 536 CE ‘dust veil’ (Larsen et al. 2008) or the ‘mystery cloud’ of 536 CE (Stothers 1984). That the initiation of this restricted cooling at 536 CE postdates the evolution of both oceanic and atmospheric influences (as indicated by low pre-536 CE values in our summation curve) demonstrates that LALIA cannot be isolated from the DACP context but may actually represent an abrupt cooling within the context of the longer DACP period. Thus, also the LIA-like regime we detect arises from the long-term DACP context represented by the slow components of the climate system. Consistent with this view, there are multiple lines of proxy and modelling evidence to suggest that the centennial-scale LIA-like climatic regimes following the post-eruption climatic events have evolved from a coupled atmosphere–ocean–sea ice North Atlantic-Arctic system (Zhong et al. 2011; Miller et al. 2012; Slawinska and Robock 2018). Possibly, such an explanation may not be needed for the multi-decadal cooling (i.e. the 536/540 CE ‘double event’) at least for the 536–545 CE interval over which the cool phase can be reproduced in model simulations by volcanic radiative forcing (Toohey et al. 2016). However, this modelling also resulted in strong positive Arctic sea ice anomalies suggesting that a possible mechanism for longer term climate response may have been reinforced by the radiative forcing anomaly i.e. the 536/540 CE ‘double event’.

The natural variability of this coupled system may represent a continuum of intermittent cold periods being spaced at roughly millennial intervals through the Pleistocene and Holocene climates (Bond et al. 2001). IRD fluctuations indeed provide the basis for several assessments of Holocene climate variability, particularly the cold episodes (Mayewski et al. 2004; Rohling and Pälike 2005; Wanner et al. 2011; Bevan et al. 2017). Solar signals in the IRD and tree-ring records, implied by the statistically significant correlations (Figs. 6c, e), as well as the volcanic signal in the temperature record (Fig. 6d), appear as further proxy-based indications of the coupled system, with an overarching role of solar activity on natural climate variability on centennial and longer scales, at least over the North Atlantic-Arctic sector. That solar and volcanic activities, but not the IRD records, showed statistically significant correlations here with terrestrial climate may arise from differing levels of autocorrelation in the tested records (see Macias-Fauria et al. 2012), in particular as the solar/volcanic forcing data comes with higher resolution than the IRD records.

These proxy limitations notwithstanding, the solar and volcanic activities are expected to impact the emergence and sustainability of the LIA-like regimes over the BCE era through the foregoing mechanisms inferred for the Common Era. The assessment of volcanic forcing becomes increasingly difficult due to uncertainties in ice-core dating prior to the 2.5 ka, after which date the sulphate signals can be confidently attributed to large eruptions (Sigl et al. 2015). A strong qualitative association between the climate and volcanic records (Kobashi et al. 2017) is remarkable even by visual inspection, with an overlapping period of cold and less cloudy climate and increased high-amplitude volcanic signals from the mid second to early first millennium BCE (marked by horizontal arrow in Fig. 6f). The early phase of this LIA-like regime could tentatively be linked with a large eruption of as yet unknown provenance resulting in dendrochronologically dated frost rings in 1627 BCE in western North American and Finnish Lapland pine chronologies (Salzer and Hughes 2007; Helama et al. 2019) (see Fig. 1). Alternatively, the timeframe of 1600–1500 BCE is punctuated by other tree-ring marker years such as 1597, 1560, 1546 and 1544 BCE (Pearson et al. 2018, 2020; Helama et al. 2019) potentially linked with the Thera (Santorini) eruption (Marinatos 1939), which may have been the largest known Holocene eruption (Johnston et al. 2014). Similar to the 536 CE event, however, these volcanic events appear to postdate the emergence of the LIA-like regime, suggesting an involvement of an additional forcing mechanism. Here, the tree-ring records show a sustained period of cold climate and reduced cloudiness between 1670 and 860 BCE (Fig. 6b). The summation curve thus shows markedly low values for a few decades prior to the volcanically triggered event, which is consistent with the initiation of the LIA-like climatic regimes during the Common Era. This 1670–860 BCE interval is also broadly coeval to an increase in the IRD%, which exceeds in magnitude the IRD anomalies recorded for the Common Era (Fig. 6e). Also, the cool poles (CP) event around 3 ka (Mayewski et al. 2004) (Table S1) appears coeval to this 1670–860 BCE event. This prolonged LIA-like regime evident from the seventeenth century BCE onwards appears have received relatively little attention from palaeoclimateologists (see, however, Avnaim-Katav et al. 2019), despite the discussions on short-term events from the NH eruptions of Aniakchak (Alaska) and Thera volcanoes (see Abbott and Davies 2012 and references therein) in the seventeenth and/or sixteenth century BCE). Here, we note the possibility that this event is also represented by the cool periods Denton and Karlén (1973) inferred from their observations on the NH glacier expansions.

The 3240–2910 BCE anomaly coincides with the ‘5.2 ka event’ recorded globally as a cold period (Magny et al. 2006) and with either wet (Magny et al. 2006; Roland et al. 2015) and/or dry (Magny et al. 2006) conditions recorded in the north-west Europe, and overlaps with the long-term development of the coinciding CP event (Mayewski et al. 2004). The LIA-like conditions in our data and the dating of the event both agree remarkably with these figures and those demonstrating an onset at 5.23 ka as averaged from a set of 43 proxy records worldwide (Magny et al. 2006; Roland et al. 2015). Similar to the LIA, both the early first millennium (1000–800 BCE) and the 3240–2910 BCE climate deteriorations contribute to peatland tree population reductions (Edvardsson et al. 2016) and human population downturns in the same region (Bevan et al. 2017). The earliest LIA-like anomaly is recorded for the 5450–5360 BCE interval which is coincident with the ‘7.4 ka’ event. Albeit less investigated, this event has been described as a cold and arid period in the Mediterranean and correlated with coincident anomalies identified in fourteen terrestrial, lacustrine and oceanic proxy records in the Alps, in and around the central and eastern Mediterranean, and in eastern Africa (Filippidi et al. 2016). Here we also note the local minima in the long-term solar forcing curve both at the 7.4 ka and 5.2 ka (Fig. 6c) and the IRD% increase predating these events. The estimates of volcanic forcing data come with increasing dating uncertainties over these BCE intervals but the peaks can be observed around 5450 BCE and 3150 BCE (Fig. 6f). These multiple lines of evidence point to common mechanisms underlying the evolution of the inferred LIA-like climatic regimes in our data and anticipate the negative human consequences not only during the LIA but at least during the ‘5.2 ka event’ and 1627 BCE and 536 CE onwards (Büntgen et al. 2016; Bevan et al. 2017).

Our interpretation with suggested mechanisms behind the LIA-like climatic regimes may be seen to concur with previous indications, with some reservations. That is, the Holocene proxy evidence for common mechanisms is reasonable, when excluding the ‘8.2 ka event’. This event was caused by the meltwater flux from remaining North American glacial lake systems into the North Atlantic (Alley and Agústdottir, 2005; Rohling and Pälike 2005), thus resulting in a widespread cold phase of distinctly different origin than those events recorded in more recent times. As an early Holocene event, it probably had more in common with the glacial world than with forcing factors of more recent Holocene events (Mayewski et al. 2004). Further, our results show that no single process could explain all LIA-like climatic regimes but a combination of factors may have played an essential role, with feedback effects. Such conclusions would in large part agree with previous interpretations of Holocene climate variability (Mayewski et al. 2004; Wanner et al. 2011), given that the ‘8.2 ka event’ and its drivers are excluded, which indeed was the case here by virtue of the temporal limitations inherent to our tree-ring proxy records (Helama et al. 2010, 2018a). The calendar dating of the events (Bond et al. 2001; Mayewski et al. 2004; Wanner et al. 2011) remains debated (Table S1) and our data was not clearly consistent with the previously suggested event dates. Noteworthy, our detection of LIA-like climatic regimes considered not only temperature but also cloud cover estimates in a fashion that mimicked the actual LIA climatic regime, as observed here for most recent pre-industrial centuries. Also, the climatic signals in our tree-ring data are representative of summer season and high-latitude north European sites. They also come with high temporal resolution while some of the abrupt events may have remained underrepresented in low-resolution proxy records of other studies. Even so, we also referred to a number of previously suggested climatic events inferred from other proxy compilations coinciding with our LIA-like climatic periods (Bond et al. 2001; Seppä et al. 2002; Berglund 2003; Mayewski et al. 2004; Magny et al. 2006; Kaufman et al. 2009; Wanner et al. 2011; Roland et al. 2015; Büntgen et al. 2016; Filippidi et al. 2016; Bevan et al. 2017; Avnaim-Katav et al. 2019).

The episodic CP events (Mayewski et al. 2004) were generally reflected in our tree-ring record, following the results from climate models suggesting north European precipitation anomalies to be attributable to the Arctic sea-ice trends; while the recent sea-ice loss is linked to a more southerly jet-stream and thus, with increased summer precipitation over the region (Screen 2013), a northward shift of the summer jet-stream steering storms away from northern Europe could be anticipated with Arctic sea-ice expansion. In addition, positive correlations between jet latitude and the NAO and negative correlations with the EAP (Hall and Hanna 2018) would agree with the foregoing suggestions of the negative EAP-phase being more prevalent during the periods of low solar forcing (Moffa-Sánchez et al. 2014) and as a precursor for the initiation of the LIA-like climatic regimes. An enhancement of blocking activity between Greenland and Western Europe typical of this EAP-phase has also been shown to result in anomalous sea-ice accumulation in the Arctic (Ionita et al. 2016). Subsequently, the resulting increase in Arctic drift ice through the Fram Strait is expected to result in a weaker AMOC (Bond et al. 2001; Ionita et al. 2016). Over decadal and even longer scales, such a sequence could explain the sustained period of colder temperatures and reduced cloudiness found here to punctuate the interglacial climate, through a combination of ocean and sea-ice feedbacks. Moreover, links to the Arctic sea-ice trends indicating increased wetness (cloudiness) due to the ongoing sea-ice loss (Screen 2013) accords with the recent increase in cloud cover reconstructed here that starts in the late nineteenth century CE and accelerates since the 1980s CE (Figs. 4a and 6a), in close agreement with the observed history of the Arctic sea ice (Polyak et al. 2010; Polyakov et al. 2012). Thus, the reversal of the LIA-like conditions since the late nineteenth century can be expected to mirror the ongoing decline in Arctic sea-ice extent.

## Conclusions

Our approach to simultaneously reconstruct the multiple constituents of LIA-like climates, with low summer temperatures occurring together with clear skies, allows a more versatile interpretation of past climate variability and its internally driven and externally forced mechanisms, compared to analyses exploring a single climate parameter. As suggested, the regime shifts were a product of a sequence where the strong volcanic forcing was predated by influences from additional, non-volcanic factors most likely resulting from reduced solar forcing generating a tendency towards a cold phase of the AMO and negative EAP and thus, as a precursor for increasing AMOC weakening and initiation of the finally volcanically triggered LIA-like climate state. This would represent pre-conditioning required prior to a volcanic eruption for the initiation of an LIA-like state. Linking the Arctic sea-ice extent specifically with hydroclimatic trends is likely to explain the sensitivity of our cloud cover record to portray anomalous conditions in connection to previously defined Holocene climatic events, especially the “cool poles” events, in accordance with expected LIA-like climate signal. This may also explain why the timing and forcing of our LIA-like periods and those published previously can also show inconsistencies, particularly when compared to the use of precisely dated multiple tree-ring proxy records that have enabled the detection of simultaneous long-lasting drops in temperature and cloud cover, diagnostics of LIA-like climate regimes in sites downstream of the North Atlantic region. Conversely, the reversal of the LIA-like conditions since the late nineteenth century reflects the decline in Arctic sea-ice extent we observe today.

## Supplementary Information

Below is the link to the electronic supplementary material.

Supplementary file1 (PDF 1305 KB)
