# Supplementary material for: Recurrent transitions to Little Ice Age-like climatic regimes over the Holocene
Source: Clim Dyn. 2021 Feb 6;56(11-12):3817–33. doi: 10.1007/s00382-021-05669-0 (PMC8550666; doi:10.1007/s00382-021-05669-0)
Supplement: Supplementary file 1 — Supplementary file1 (PDF 1305 KB) [file 382_2021_5669_MOESM1_ESM.pdf]

Recurrent transitions to Little Ice Age-like climatic regimes over the Holocene

Supplementary Items

Content

- Figure S1: Palaeoclimate reconstruction of summer (June–August) temperatures
- Figure S2: Palaeoclimate reconstruction of summer (JJA) cloud cover variations
- Figure S3: Palaeoclimate reconstruction of summer (July) temperatures
- Figure S4: Normalisation of the data prior to the superposed epoch analysis
- Figure S5: Sea level pressure data and the teleconnection indices
- Figure S6: Superposed epoch analysis for the adjusted data of the Common Era
- Figure S7: Lagged correlations between the solar forcing and climate data
- Figure S8: Pearson correlations between the proxy records
- Figure S9: Spatial correlations with the index of East Atlantic Pattern
- Figure S10: Cloud cover variations during the sixth century CE

- Table S1: Expected cold climate events of the past 7500 years
- Table S2: Online availability of the data
- Table S3: Global volcanic forcing of the Common Era
- Table S4: Time-series of cloud cover record

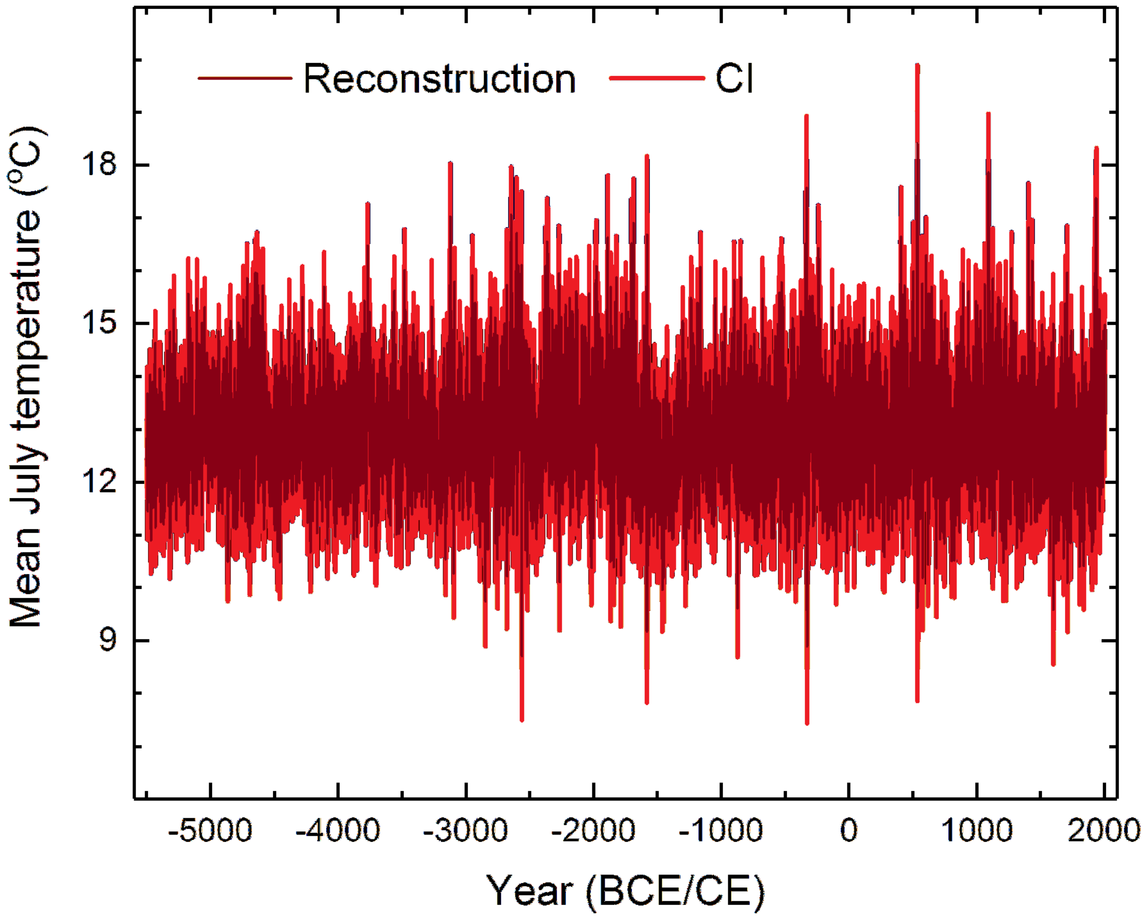

Fig. S1. Palaeoclimate reconstruction of summer (July) temperatures. This reconstruction is based on the tree-ring width data, shown with the 95% confidence intervals (CI) of the reconstruction (Helama et al. 2010). The underlying data originates from several previous tree-ring analyses (Eronen et al. 1999, 2002; Helama et al. 2005, 2008).

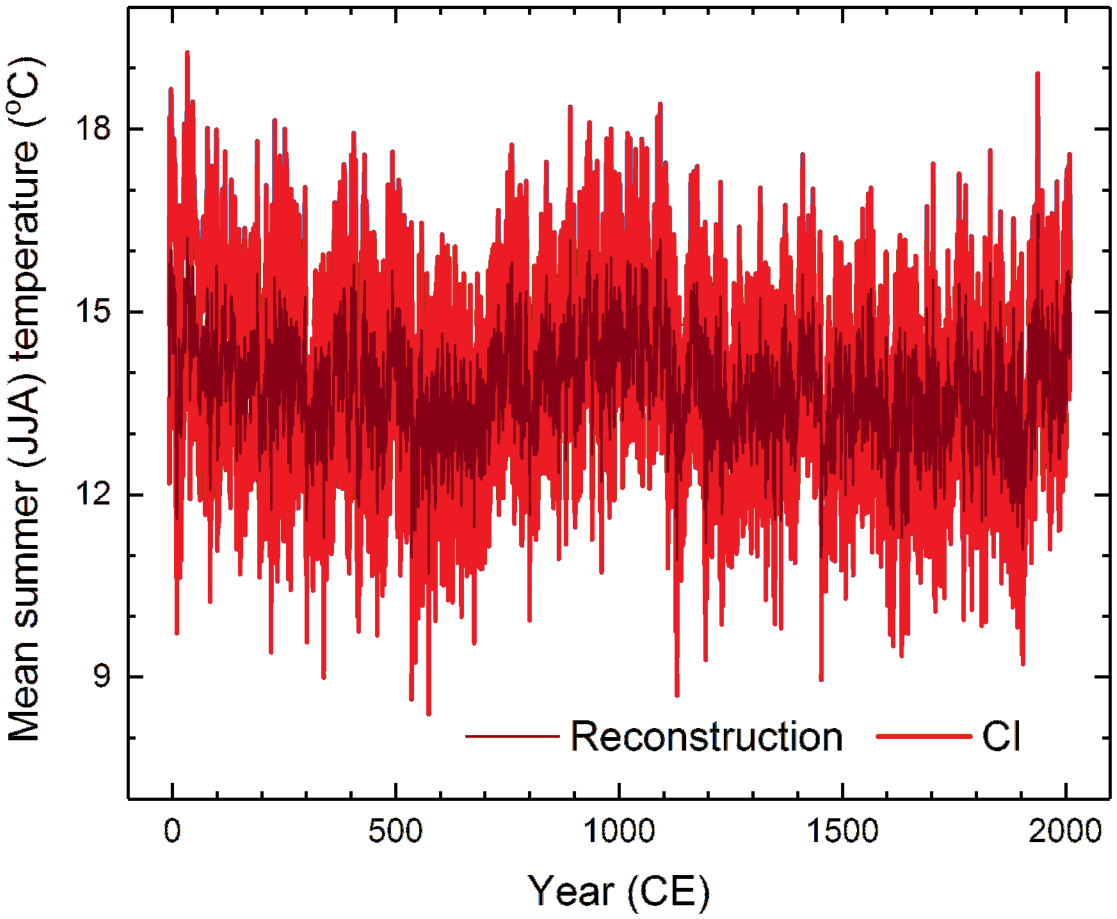

Fig. S2. Palaeoclimate reconstruction of summer (June-August) temperatures. This reconstruction is based on the maximum latewood density data, shown with the 95% confidence intervals (CI) of the reconstruction (Matskovsky and Helama 2014). The underlying data originates from several previous tree-ring analyses (Schweingruber et al. 1988; Grudd 2008; Melvin et al. 2013; Esper et al. 2012).

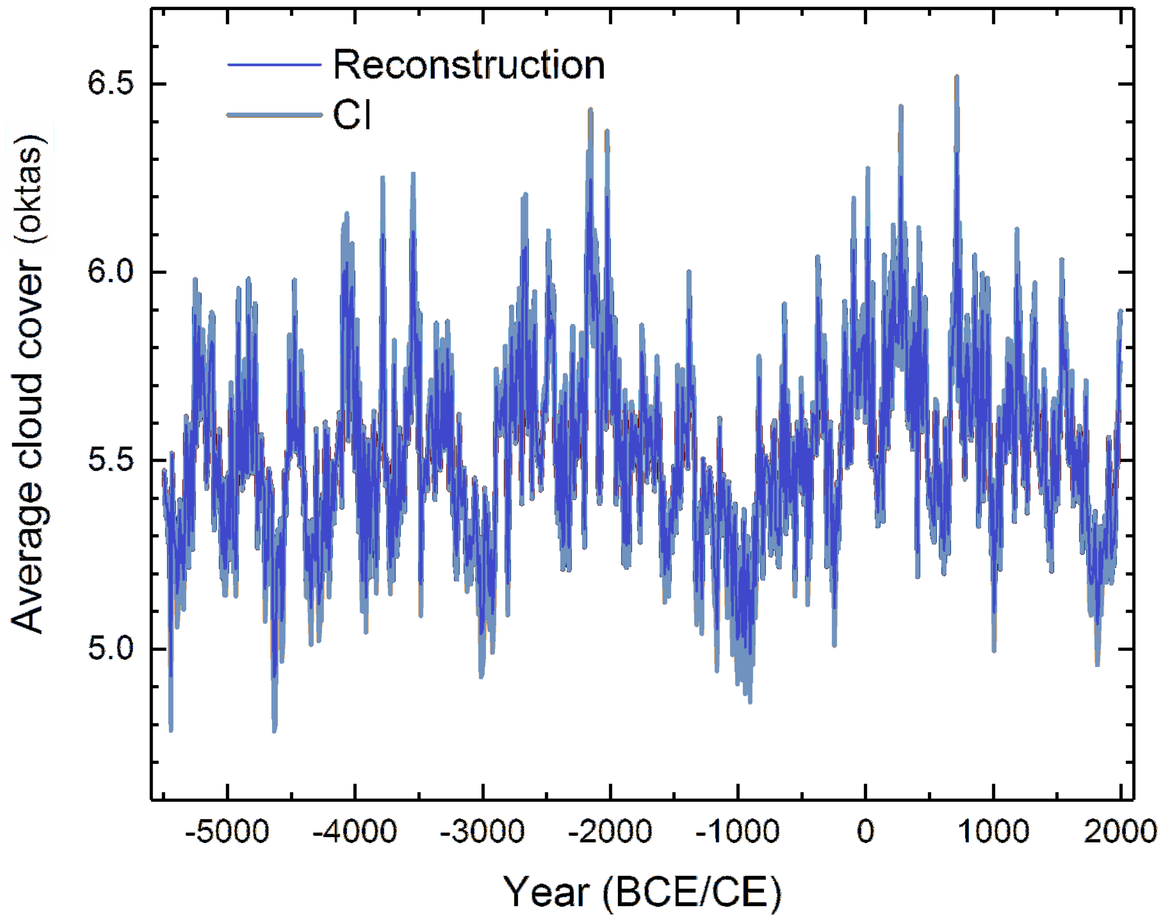

Fig. S3. Palaeoclimate reconstruction of average summer (June-August) cloud cover variations. This reconstruction is based on the decadal tree-ring $\delta^{13}C$ data, shown with the 95% confidence intervals (CI) of the reconstruction (Helama et al. 2018a).

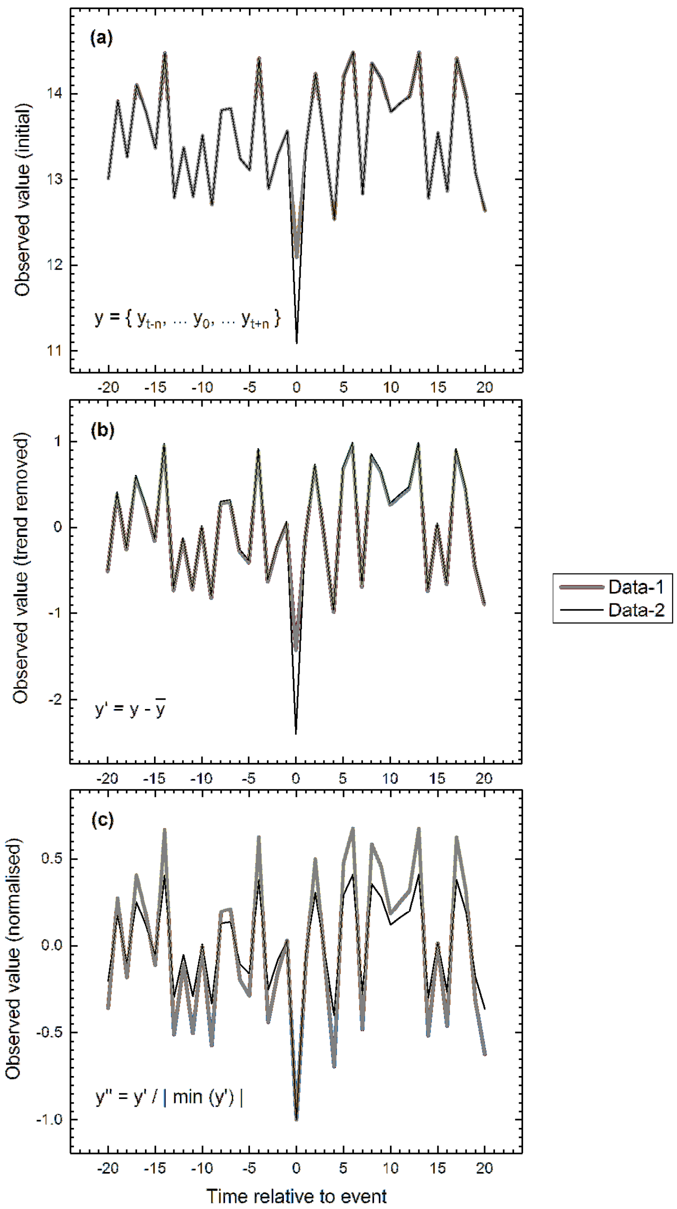

Fig. S4. Normalisation of the data prior to the superposed epoch analysis. A series of surrogate data without (Data-1) and with a disproportionate extreme value (Data-2) (a) are normalised by removing the mean in the window (y_{t-n}, … y_0, … y_{t+n}) (b) after which each value is divided by the minimum absolute value in that window (c).

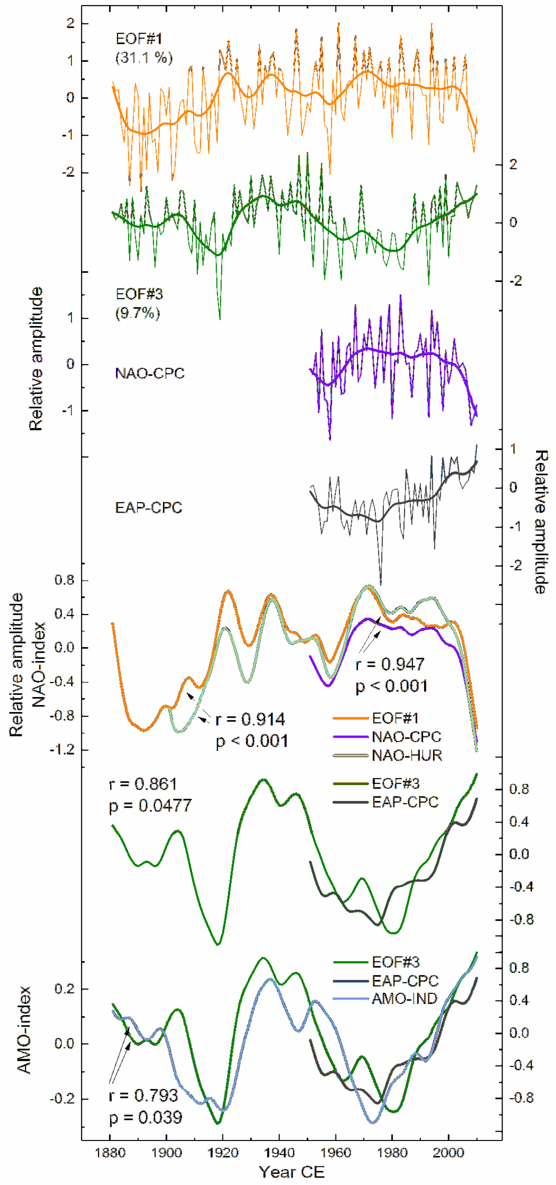

Fig. S5. Sea level pressure data and the teleconnection indices. The EOFs of the sea level pressure (SLP) data (1881-2010 CE) (Compo et al. 2011) explaining 24.0% and 14.3% of the SLP variance and corresponding to the teleconnection indices of the North Atlantic Oscillation (NAO-CPC), the East Atlantic Pattern (EAP-CPC) (Barnston and Livezey 1987) (1951-2010 CE) and the longer NAO-index series (NAO-HUR) (Hurrell and Deser 2010) (1901-2010 CE). These indices, and those of the Atlantic Multidecadal Oscillation (AMO) (Enfield et al. 2001), were smoothed with 15-year spline function (thick curves) to correspond with time scales relevant to our tree-ring proxy data. Pearson correlation coefficients (r) were calculated between the smoothed series. Statistical significance (p) was assessed using one hundred thousand Monte Carlo simulations. All the data is shown for the summer season (June through August).

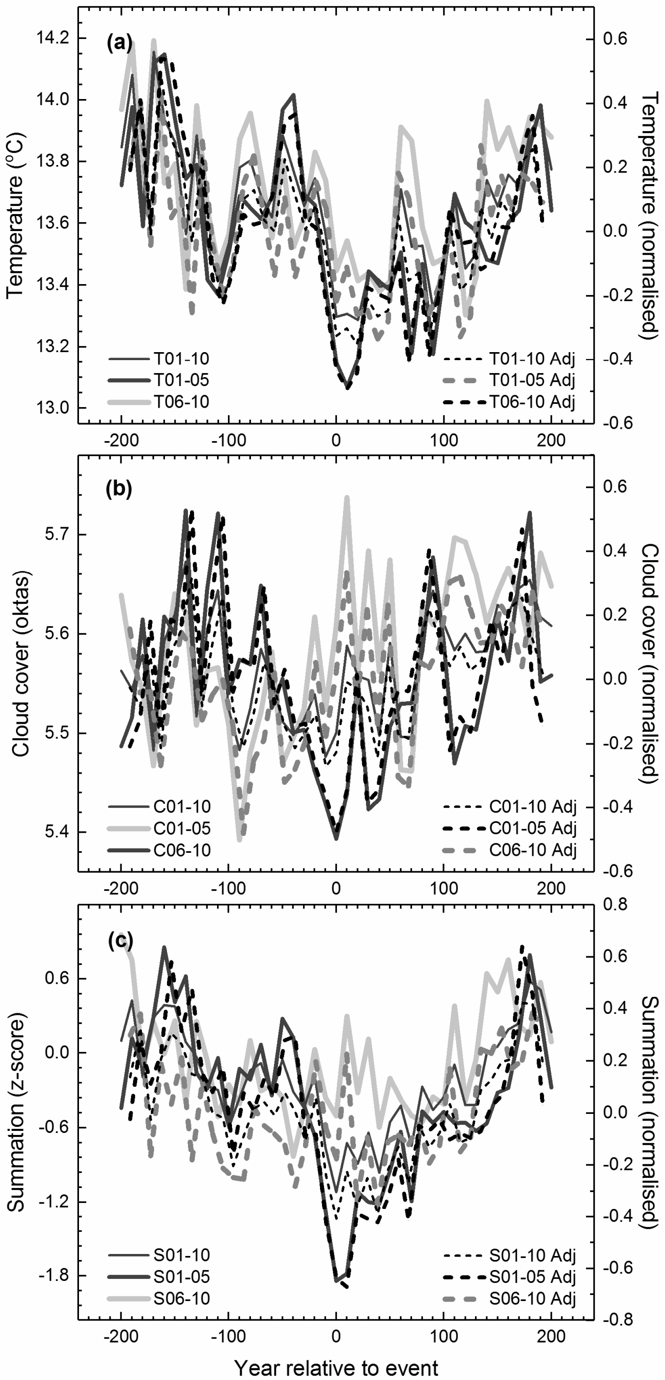

Fig. S6. Superposed epoch analysis (SEA). Temperature (a), cloud cover (b) and the summation data (c) centred on ten (T01-10, C01-10 and S01-10) and five (T01-05, C01-05 and S01-05) largest (and five next largest (T06-10, C06-10 and S06-10)) volcanic eruptions of the Common Era (Sigl et al. 2015) (see Table S3). Alternative SEAs were produced for initial (non-normalised) and normalised data (Adj) in windows. The SEAs produced using non-normalised data are also shown in Fig. 5.

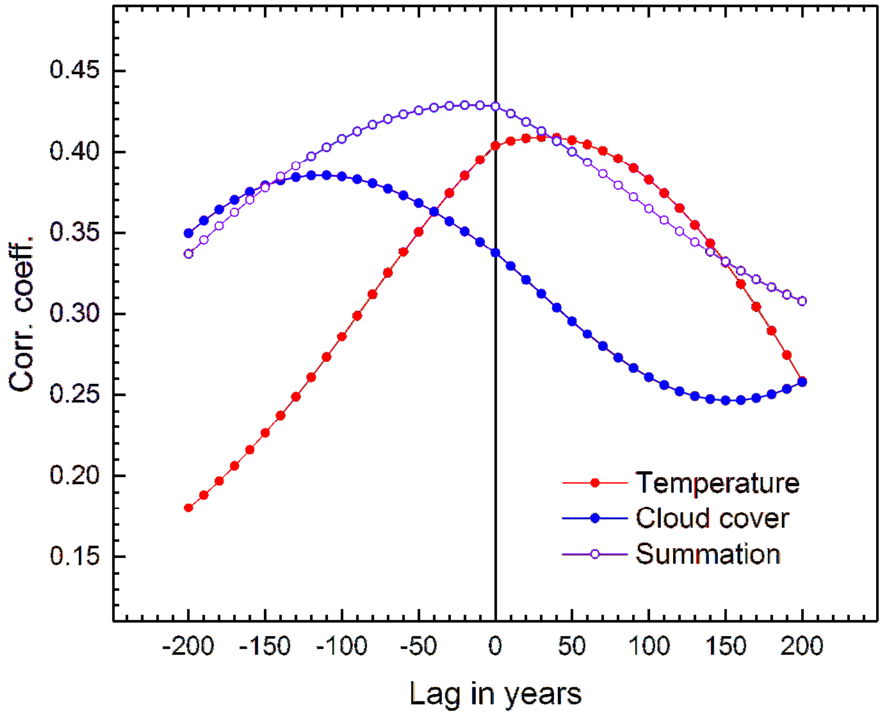

Fig. S7. Lagged correlations between the solar forcing and climate data. Pearson correlation coefficients calculated between the reconstruction of total solar irradiance (Steinhilber et al. 2009) and the temperature, cloud cover and summation data of this study (see Fig. 6c) over the past 7.5 thousand years. Positive (negative) lag indicates that the solar forcing predates (postdates) climate data.

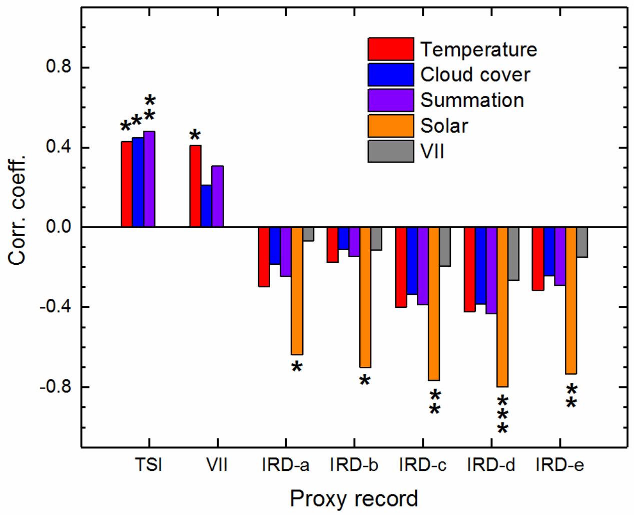

Fig. S8. Pearson correlations between the proxy records. Correlations are shown between the temperature, cloud cover and summation data of this study and the records of total solar irradiance (TSI) (Steinhilber et al. 2009) and the volcanic impact index (VII) (Kobashi et al. 2017), and those of North Atlantic ice-rafted debris (Bond et al. 2001) including the percentage of hematite-stained grains in the multicore MC52-VM29-191 record (IRD-a), the percentage of Icelandic glass in the same record (IRD-b) and that in the multicore MC21-GGC22 record (IRD-c), the percentage of detrital carbonate in the multicore MC21-GGC22 record (IRD-d), and the stack of the percentage records (IRD-e). All the records were filtered using the spline functions corresponding to 1000-year rigidity (see Fig. 6d) and the correlations calculated over the past 7.5 thousand years. Statistical significance at levels $p < 0.05$, $p < 0.01$ and $p < 0.001$ are denoted by one (*), two (**) and three two asterisks (**), respectively, assessed using one hundred thousand Monte Carlo simulations.

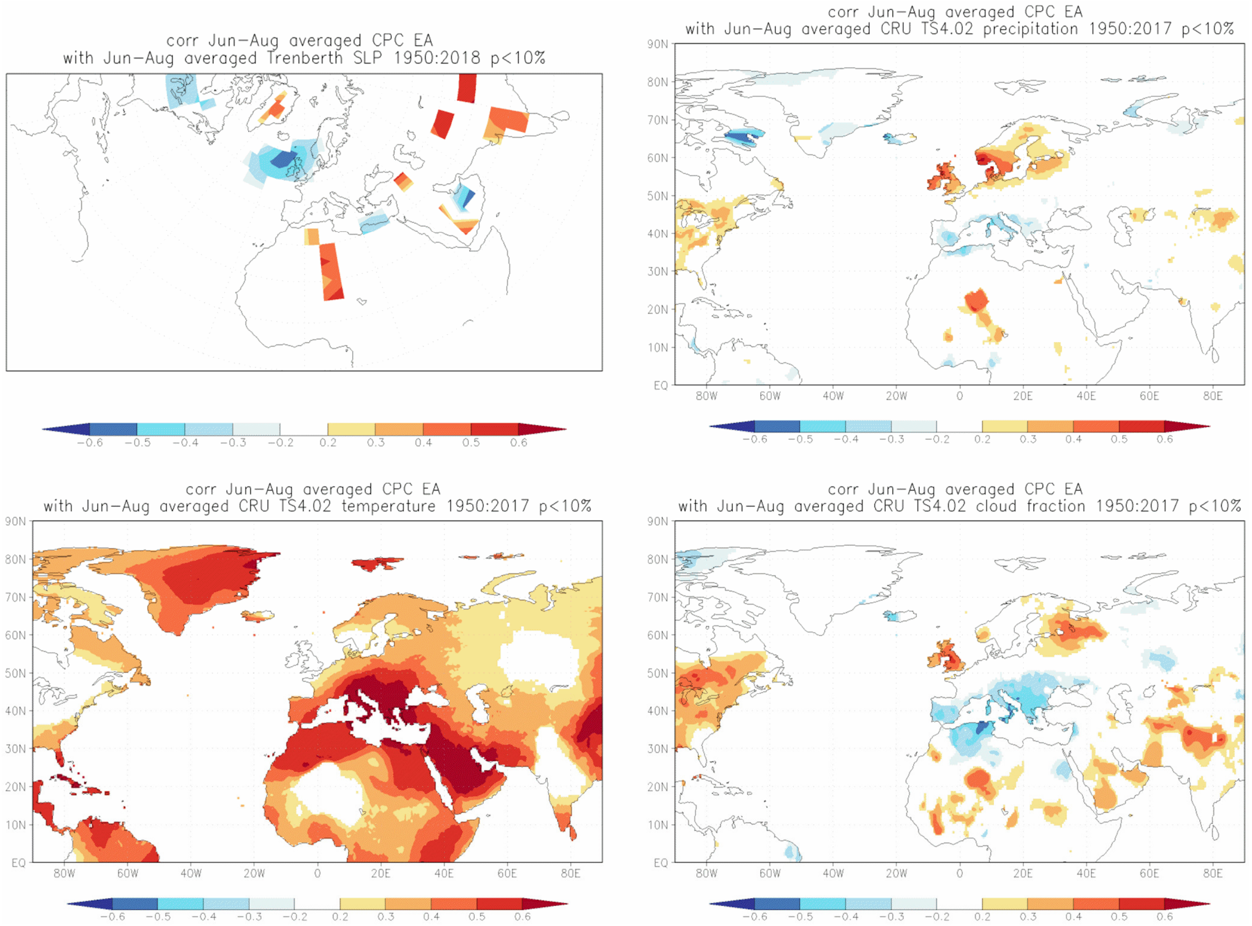

Fig. S9. Spatial correlations with the index of East Atlantic Pattern. Correlations are calculated between the index of the East Atlantic pattern (Barnston and Livezey 1987) and the data of sea level pressure (Trenberth and Paolino 1980), temperature, precipitation and cloud cover (Harris et al. 2014) and East Atlantic pattern produced at the https://climexp.knmi.nl/ (Trouet and van Oldenborgh 2013). All the data used is for the summer season (June through August). Only significant ($p < 0.1$) correlations are shown.

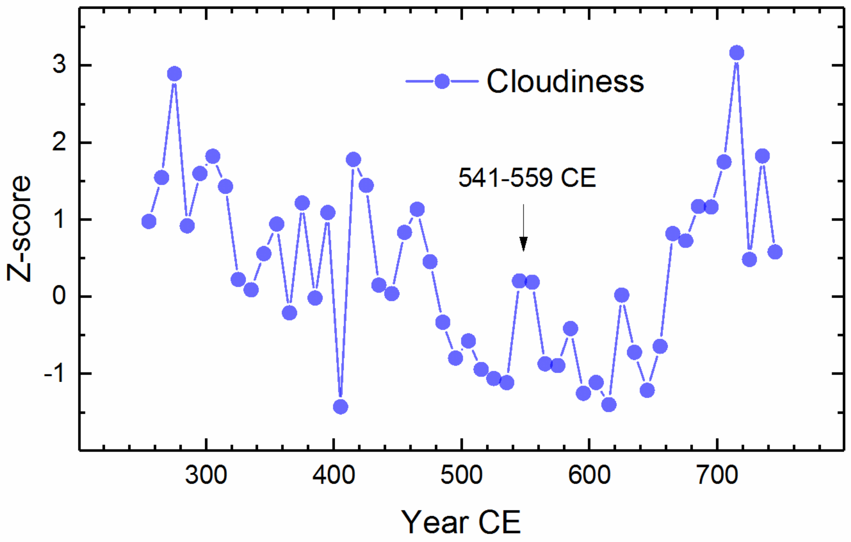

Fig. S10. Cloud cover variations during the sixth century CE. Reconstructed variations between 250 and 750 CE (Helama et al. 2018a) and the short-lived increase in overcast in the 540s and 550s CE (vertical arrow) illustrating the 'dust veil' effect discussed in more details in Helama et al. (2018b).

Table S1. Expected cold climate events of the past 7500 years. The events are given as the North Atlantic ice-rafted debris events (IRD) (Bond et al. 1997, 1999, 2001), the "cool poles" (CP) (Mayewski et al. 2004), and the Holocene cold events (HC) (Wanner et al. 2011) with their datings given in the original references. For the most recent IRD, supposedly overlapping with the Little Ice Age, we use the mid-years of the range ("between early 1600 and late 1800 A.D.") given in Bond et al. (1999). For the most recent "cool poles" event, the mid-year of the range from 600 to 150 cal. BP (rounded to decadal value) given in Mayewski et al. (2004) was used.

Event	Dating	Timeline
IRD	1750	CE
IRD	1400	cal. BP
IRD	2800	cal. BP
IRD	4200	cal. BP
IRD	5900	cal. BP
CP	380	cal. BP
CP	1100	cal. BP
CP	3000	cal. BP
CP	4000	cal. BP
CP	5500	cal. BP
HC	550	cal. BP
HC	1550	cal. BP
HC	2700	cal. BP
HC	4700	cal. BP
HC	6300	cal. BP

Table S2. Online availability of the data. The palaeoclimate and instrumental data used in this study includes the temperature reconstructions based on the tree-ring width (TRW) (Helama et al. 2010) and maximum latewood density (MXD) (Matskovsky and Helama 2014) chronologies, the reconstruction of cloud cover variability based on the stable carbon isotope ratios ($\delta^{13}C$) (Helama et al. 2018a), the reconstruction of total solar irradiance (ΔTSI) (Steinhilber et al. 2009), the volcanic impact index (VII) (Kobashi et al. 2017) and with the records of North Atlantic ice-rafted debris (IRD) (Bond et al. 2001), the Northern Hemisphere and global temperature data from the Climatic Research Unit (CRUTEM4) (Jones et al. 2012), the teleconnection indices by the Climate Prediction Center (CPC) of the North Atlantic Oscillation (NAO-CPC), East Atlantic Pattern (EAP-CPC) and Scandinavian Pattern (SCA-CPC), derived from 500 hPa GPH anomalies (Barnston and Livezey 1987), the longer NAO-index series (Hurrell and Deser 2010) (NAO-HUR), the index of the Atlantic Multidecadal Oscillation (AMO) (Enfield et al. 2001), and the sea level pressure (SLP) data (Compo et al. 2011) used for the EOF-based circulation indices.

Data	Online availability
MXD	https://www.clim-past.net/10/1473/2014/
TRW	http://www.helsinki.fi/science/dendro/data/DATA-Finland-Dendro-team.xls
$\delta^{13}C$	Table S4
ΔTSI	ftp://ftp.ncdc.noaa.gov/pub/data/paleo/climate_forcing/solar_variability/steinhilber2009tsi.txt
VII	https://www.nature.com/articles/s41598-017-01451-7#Sec8
IRD	ftp://ftp.ncdc.noaa.gov/pub/data/paleo/contributions_by_author/bond2001/bond2001.txt
CRUTEM4	https://crudata.uea.ac.uk/cru/data/temperature/
NAO-CPC	ftp://ftp.cpc.ncep.noaa.gov/wd52dg/data/indices/nao_index.tim
EAP-CPC	ftp://ftp.cpc.ncep.noaa.gov/wd52dg/data/indices/ea_index.tim
SCA-CPC	ftp://ftp.cpc.ncep.noaa.gov/wd52dg/data/indices/scand_index.tim
NAO-HUR	https://climatedataguide.ucar.edu/climate-data/hurrell-north-atlantic-oscillation-nao-index-pc-based
AMO	https://www.esrl.noaa.gov/psd/data/timeseries/AMO/
SLP	https://rda.ucar.edu/datasets/ds131.1/

Table S3. Global volcanic forcing of the Common Era. The largest events of volcanic forcing (GVF; Wm^{-2}) (Sigl et al. 2015) since 1 CE are given with their calendar years (CE) according to their forcing estimates belonging to the groups of five (v) and ten (x) largest eruptions used in the superposed epoch analysis. Notes for the eruptions discussed in the text: the 1250s CE eruption for which the forcing is estimated to peak in 1258 and 1259 CE occurred in 1257 CE (Stoffel et al. 2015) and that date is used in the text; the enigmatic dating of the 1450s CE eruption (occurring either in 1452/1453 or 1458 CE) (Plummer et al. 2012) does not influence our decadal analyses and is simply referred to as the 1450's eruption in the text; the 540s CE eruption is often discussed together with the post-eruption climatic event in 536 CE and in the context of the mid-sixth century climate anomalies (e.g. Sigl et al. 2015) and this volcanic event (i.e. 536/540 CE) is similarly referred to in the text.

Rank	CE	GVF	Group
1	1258	-32.79	v, x
2	1458	-20.55	v, x
3	540	-19.14	v, x
4	1815	-17.20	v, x
5	1230	-15.90	v, x
6	1783	-15.49	x
7	682	-15.43	x
8	574	-14.49	x
9	266	-14.46	x
10	1809	-12.01	x

Table S4. Time-series of cloud cover record. Reconstructed cloud cover values (Helama et al. 2018a) (oktas) with their Monte Carlo (see Macias-Fauria et al. 2012) based estimates of confidence intervals (CI). Calendar years (positive values for CE and negative for BCE) refer to mid years of the decades.

Year CE/BCE	Cloud cover	CI 95%	CI 95%	CI 99%	CI 99%
-5495	5.468	5.459	5.474	5.457	5.476
-5485	5.384	5.354	5.406	5.349	5.412
-5475	5.386	5.356	5.408	5.351	5.413
-5465	5.327	5.283	5.360	5.275	5.368
-5455	5.361	5.325	5.388	5.319	5.394
-5445	4.928	4.784	5.035	4.759	5.060
-5435	5.517	5.514	5.520	5.514	5.521
-5425	5.328	5.284	5.361	5.277	5.369
-5415	5.280	5.225	5.322	5.215	5.332
-5405	5.265	5.205	5.309	5.194	5.320
-5395	5.148	5.059	5.214	5.043	5.230
-5385	5.193	5.115	5.251	5.102	5.264
-5375	5.369	5.336	5.394	5.330	5.400
-5365	5.230	5.161	5.281	5.149	5.293
-5355	5.301	5.251	5.339	5.242	5.348
-5345	5.185	5.106	5.245	5.092	5.259
-5335	5.326	5.282	5.360	5.274	5.367
-5325	5.595	5.578	5.617	5.574	5.621
-5315	5.528	5.523	5.534	5.522	5.535
-5305	5.275	5.218	5.318	5.208	5.328
-5295	5.422	5.401	5.437	5.398	5.441
-5285	5.578	5.564	5.597	5.561	5.600
-5275	5.314	5.266	5.349	5.258	5.358
-5265	5.538	5.532	5.546	5.530	5.548
-5255	5.886	5.815	5.982	5.798	5.998
-5245	5.544	5.537	5.555	5.535	5.556
-5235	5.742	5.697	5.801	5.687	5.812
-5225	5.855	5.789	5.942	5.774	5.958
-5215	5.634	5.609	5.666	5.604	5.672
-5205	5.750	5.704	5.812	5.693	5.822
-5195	5.778	5.727	5.847	5.715	5.859
-5185	5.695	5.659	5.742	5.651	5.750
-5175	5.623	5.601	5.653	5.596	5.658
-5165	5.442	5.427	5.454	5.424	5.456
-5155	5.502	5.501	5.502	5.501	5.502
-5145	5.718	5.678	5.771	5.668	5.780
-5135	5.772	5.722	5.839	5.710	5.851
-5125	5.816	5.757	5.894	5.744	5.907

-5115	5.808	5.751	5.884	5.738	5.897
-5105	5.353	5.316	5.382	5.309	5.388
-5095	5.518	5.515	5.521	5.515	5.522
-5085	5.488	5.484	5.491	5.484	5.492
-5075	5.403	5.378	5.422	5.373	5.426
-5065	5.568	5.556	5.583	5.553	5.586
-5055	5.369	5.336	5.394	5.330	5.400
-5045	5.253	5.191	5.300	5.180	5.311
-5035	5.440	5.424	5.452	5.422	5.455
-5025	5.215	5.143	5.269	5.130	5.282
-5015	5.215	5.143	5.269	5.130	5.281
-5005	5.326	5.282	5.359	5.274	5.367
-4995	5.459	5.448	5.467	5.446	5.469
-4985	5.292	5.239	5.332	5.230	5.341
-4975	5.666	5.636	5.707	5.629	5.714
-4965	5.606	5.587	5.631	5.582	5.636
-4955	5.266	5.207	5.311	5.197	5.321
-4945	5.300	5.249	5.338	5.241	5.347
-4935	5.213	5.140	5.267	5.127	5.280
-4925	5.618	5.597	5.647	5.592	5.652
-4915	5.866	5.798	5.956	5.782	5.972
-4905	5.476	5.469	5.481	5.468	5.482
-4895	5.633	5.609	5.666	5.603	5.671
-4885	5.439	5.422	5.451	5.420	5.454
-4875	5.471	5.463	5.477	5.462	5.479
-4865	5.715	5.676	5.768	5.666	5.777
-4855	5.469	5.460	5.475	5.459	5.477
-4845	5.467	5.457	5.474	5.456	5.475
-4835	5.887	5.815	5.982	5.798	5.999
-4825	5.652	5.624	5.689	5.618	5.696
-4815	5.562	5.551	5.577	5.549	5.579
-4805	5.474	5.467	5.480	5.466	5.481
-4795	5.716	5.676	5.769	5.667	5.778
-4785	5.834	5.773	5.917	5.758	5.931
-4775	5.600	5.582	5.624	5.578	5.629
-4765	5.316	5.269	5.351	5.261	5.359
-4755	5.470	5.461	5.476	5.459	5.477
-4745	5.395	5.368	5.415	5.363	5.420
-4735	5.556	5.546	5.569	5.544	5.572
-4725	5.449	5.435	5.459	5.433	5.461
-4715	5.431	5.413	5.445	5.410	5.448
-4705	5.159	5.073	5.224	5.058	5.239

-4695	5.288	5.234	5.328	5.224	5.337
-4685	5.237	5.171	5.287	5.159	5.299
-4675	5.473	5.466	5.479	5.464	5.480
-4665	5.415	5.393	5.432	5.389	5.436
-4655	5.417	5.395	5.433	5.391	5.437
-4645	5.069	4.960	5.150	4.941	5.169
-4635	4.926	4.782	5.034	4.756	5.059
-4625	4.939	4.798	5.044	4.773	5.069
-4615	5.241	5.176	5.290	5.164	5.302
-4605	5.273	5.215	5.316	5.205	5.326
-4595	5.217	5.146	5.271	5.133	5.283
-4585	5.307	5.258	5.344	5.250	5.353
-4575	5.075	4.968	5.155	4.949	5.174
-4565	5.116	5.020	5.189	5.003	5.206
-4555	5.467	5.458	5.474	5.456	5.475
-4545	5.385	5.355	5.407	5.350	5.412
-4535	5.521	5.518	5.525	5.517	5.526
-4525	5.485	5.481	5.489	5.480	5.490
-4515	5.767	5.718	5.833	5.706	5.844
-4505	5.513	5.511	5.515	5.511	5.515
-4495	5.609	5.590	5.636	5.585	5.640
-4485	5.555	5.545	5.567	5.543	5.570
-4475	5.884	5.813	5.979	5.796	5.995
-4465	5.529	5.524	5.535	5.523	5.536
-4455	5.588	5.572	5.609	5.568	5.612
-4445	5.563	5.552	5.578	5.549	5.580
-4435	5.489	5.486	5.492	5.485	5.493
-4425	5.734	5.691	5.791	5.681	5.801
-4415	5.665	5.635	5.705	5.627	5.712
-4405	5.387	5.357	5.408	5.352	5.414
-4395	5.461	5.451	5.469	5.449	5.471
-4385	5.213	5.141	5.268	5.128	5.280
-4375	5.210	5.137	5.265	5.124	5.278
-4365	5.298	5.247	5.337	5.238	5.345
-4355	5.189	5.111	5.248	5.097	5.262
-4345	5.110	5.012	5.184	4.995	5.201
-4335	5.305	5.255	5.342	5.246	5.351
-4325	5.278	5.222	5.320	5.212	5.330
-4315	5.236	5.169	5.286	5.157	5.298
-4305	5.569	5.557	5.586	5.554	5.589
-4295	5.274	5.217	5.317	5.207	5.327
-4285	5.119	5.023	5.191	5.006	5.207

-4275	5.158	5.072	5.223	5.057	5.238
-4265	5.160	5.075	5.225	5.060	5.239
-4255	5.392	5.364	5.413	5.359	5.418
-4245	5.288	5.234	5.328	5.224	5.337
-4235	5.583	5.568	5.602	5.564	5.606
-4225	5.269	5.210	5.313	5.200	5.323
-4215	5.567	5.555	5.583	5.552	5.585
-4205	5.211	5.138	5.266	5.125	5.279
-4195	5.327	5.283	5.360	5.275	5.368
-4185	5.386	5.356	5.408	5.351	5.413
-4175	5.497	5.495	5.498	5.495	5.498
-4165	5.368	5.334	5.393	5.328	5.399
-4155	5.482	5.477	5.486	5.476	5.487
-4145	5.507	5.506	5.507	5.506	5.507
-4135	5.547	5.539	5.557	5.537	5.559
-4125	5.603	5.584	5.627	5.580	5.631
-4115	5.520	5.517	5.524	5.516	5.524
-4105	5.402	5.377	5.421	5.372	5.426
-4095	5.992	5.901	6.114	5.879	6.135
-4085	6.004	5.911	6.129	5.889	6.151
-4075	5.884	5.813	5.979	5.796	5.995
-4065	6.026	5.929	6.157	5.906	6.180
-4055	5.563	5.552	5.578	5.549	5.580
-4045	5.814	5.756	5.891	5.742	5.905
-4035	5.944	5.862	6.054	5.843	6.073
-4025	5.961	5.876	6.075	5.856	6.095
-4015	5.764	5.716	5.829	5.704	5.841
-4005	5.479	5.473	5.484	5.472	5.485
-3995	5.637	5.612	5.670	5.606	5.676
-3985	5.801	5.745	5.875	5.732	5.888
-3975	5.350	5.312	5.379	5.305	5.385
-3965	5.666	5.636	5.707	5.629	5.714
-3955	5.334	5.292	5.366	5.284	5.373
-3945	5.181	5.101	5.241	5.087	5.255
-3935	5.573	5.560	5.590	5.557	5.593
-3925	5.506	5.506	5.507	5.505	5.507
-3915	5.137	5.045	5.205	5.029	5.221
-3905	5.391	5.363	5.412	5.358	5.417
-3895	5.544	5.537	5.554	5.535	5.556
-3885	5.255	5.193	5.301	5.182	5.312
-3875	5.394	5.367	5.414	5.362	5.419
-3865	5.456	5.445	5.465	5.443	5.467

-3855	5.607	5.588	5.633	5.583	5.637
-3845	5.459	5.447	5.467	5.446	5.469
-3835	5.219	5.149	5.273	5.136	5.285
-3825	5.426	5.407	5.441	5.404	5.444
-3815	5.505	5.504	5.505	5.504	5.505
-3805	5.564	5.553	5.579	5.550	5.582
-3795	5.669	5.638	5.710	5.631	5.717
-3785	6.102	5.990	6.251	5.964	6.277
-3775	5.848	5.783	5.934	5.768	5.949
-3765	5.448	5.434	5.458	5.431	5.461
-3755	5.506	5.506	5.507	5.505	5.507
-3745	5.486	5.481	5.489	5.480	5.490
-3735	5.298	5.247	5.337	5.238	5.346
-3725	5.217	5.146	5.271	5.133	5.283
-3715	5.449	5.436	5.460	5.434	5.462
-3705	5.246	5.182	5.294	5.170	5.305
-3695	5.756	5.709	5.819	5.698	5.830
-3685	5.340	5.299	5.370	5.292	5.378
-3675	5.298	5.247	5.337	5.238	5.345
-3665	5.344	5.304	5.374	5.297	5.381
-3655	5.571	5.558	5.588	5.555	5.591
-3645	5.369	5.335	5.394	5.329	5.400
-3635	5.431	5.413	5.444	5.409	5.448
-3625	5.538	5.532	5.547	5.530	5.548
-3615	5.392	5.364	5.413	5.359	5.418
-3605	5.695	5.659	5.743	5.651	5.751
-3595	5.647	5.620	5.682	5.614	5.689
-3585	5.557	5.547	5.570	5.544	5.572
-3575	5.749	5.703	5.810	5.693	5.821
-3565	5.624	5.602	5.654	5.596	5.659
-3555	6.022	5.925	6.151	5.903	6.174
-3545	6.109	5.996	6.260	5.970	6.287
-3535	5.894	5.821	5.992	5.804	6.009
-3525	5.720	5.679	5.774	5.670	5.783
-3515	5.834	5.772	5.917	5.758	5.931
-3505	5.530	5.525	5.536	5.524	5.537
-3495	5.814	5.756	5.891	5.742	5.905
-3485	5.172	5.089	5.234	5.074	5.248
-3475	5.431	5.413	5.444	5.409	5.448
-3465	5.416	5.394	5.432	5.390	5.436
-3455	5.503	5.502	5.503	5.502	5.503
-3445	5.498	5.497	5.499	5.497	5.499

-3435	5.437	5.420	5.449	5.417	5.452
-3425	5.610	5.590	5.637	5.586	5.641
-3415	5.637	5.612	5.670	5.606	5.676
-3405	5.672	5.641	5.714	5.633	5.722
-3395	5.583	5.568	5.603	5.565	5.606
-3385	5.499	5.498	5.500	5.498	5.500
-3375	5.418	5.396	5.434	5.392	5.437
-3365	5.793	5.739	5.865	5.726	5.878
-3355	5.684	5.650	5.729	5.642	5.736
-3345	5.492	5.489	5.494	5.488	5.495
-3335	5.481	5.476	5.485	5.475	5.486
-3325	5.611	5.591	5.638	5.586	5.643
-3315	5.757	5.710	5.820	5.699	5.831
-3305	5.532	5.527	5.539	5.526	5.541
-3295	5.678	5.645	5.721	5.638	5.729
-3285	5.537	5.531	5.545	5.529	5.547
-3275	5.797	5.742	5.870	5.729	5.883
-3265	5.440	5.424	5.452	5.421	5.455
-3255	5.596	5.579	5.620	5.575	5.624
-3245	5.725	5.683	5.780	5.674	5.789
-3235	5.387	5.358	5.409	5.353	5.414
-3225	5.451	5.438	5.461	5.436	5.463
-3215	5.280	5.224	5.322	5.214	5.331
-3205	5.249	5.185	5.297	5.174	5.308
-3195	5.397	5.371	5.417	5.366	5.422
-3185	5.599	5.581	5.622	5.577	5.627
-3175	5.416	5.394	5.432	5.390	5.436
-3165	5.379	5.348	5.402	5.343	5.408
-3155	5.396	5.369	5.416	5.364	5.421
-3145	5.475	5.468	5.480	5.467	5.482
-3135	5.311	5.263	5.347	5.255	5.356
-3125	5.222	5.152	5.275	5.140	5.287
-3115	5.248	5.184	5.296	5.173	5.307
-3105	5.301	5.251	5.339	5.242	5.348
-3095	5.239	5.173	5.288	5.161	5.300
-3085	5.298	5.246	5.336	5.237	5.345
-3075	5.281	5.225	5.323	5.216	5.332
-3065	5.442	5.427	5.454	5.425	5.457
-3055	5.415	5.392	5.431	5.389	5.435
-3045	5.305	5.256	5.342	5.247	5.351
-3035	5.176	5.094	5.237	5.079	5.251
-3025	5.248	5.184	5.296	5.173	5.307

-3015	5.040	4.925	5.127	4.904	5.147
-3005	5.047	4.933	5.132	4.913	5.152
-2995	5.086	4.981	5.164	4.963	5.182
-2985	5.411	5.387	5.428	5.383	5.432
-2975	5.291	5.237	5.330	5.228	5.340
-2965	5.358	5.322	5.385	5.316	5.392
-2955	5.170	5.086	5.232	5.072	5.247
-2945	5.142	5.052	5.210	5.036	5.225
-2935	5.264	5.204	5.308	5.193	5.319
-2925	5.094	4.992	5.170	4.974	5.188
-2915	5.153	5.066	5.219	5.051	5.234
-2905	5.314	5.267	5.350	5.258	5.358
-2895	5.695	5.660	5.743	5.651	5.752
-2885	5.611	5.591	5.638	5.586	5.642
-2875	5.604	5.585	5.629	5.581	5.633
-2865	5.583	5.569	5.603	5.565	5.607
-2855	5.652	5.624	5.689	5.618	5.695
-2845	5.669	5.638	5.710	5.631	5.718
-2835	5.593	5.576	5.615	5.572	5.619
-2825	5.744	5.699	5.803	5.688	5.814
-2815	5.511	5.509	5.512	5.509	5.513
-2805	5.173	5.091	5.235	5.076	5.249
-2795	5.442	5.426	5.453	5.424	5.456
-2785	5.653	5.625	5.690	5.618	5.697
-2775	5.827	5.767	5.908	5.753	5.922
-2765	5.606	5.587	5.632	5.582	5.636
-2755	5.705	5.668	5.755	5.659	5.764
-2745	5.556	5.546	5.569	5.544	5.572
-2735	5.793	5.739	5.865	5.726	5.878
-2725	5.719	5.679	5.773	5.670	5.783
-2715	5.867	5.799	5.957	5.783	5.973
-2705	5.623	5.601	5.653	5.596	5.658
-2695	5.409	5.386	5.427	5.382	5.431
-2685	6.057	5.954	6.195	5.930	6.220
-2675	6.050	5.948	6.186	5.924	6.210
-2665	6.067	5.961	6.207	5.937	6.232
-2655	5.700	5.664	5.749	5.655	5.758
-2645	5.587	5.571	5.608	5.568	5.611
-2635	5.818	5.759	5.896	5.745	5.910
-2625	5.695	5.660	5.743	5.651	5.752
-2615	5.420	5.400	5.436	5.396	5.440
-2605	5.800	5.745	5.874	5.732	5.887

-2595	5.860	5.794	5.949	5.778	5.965
-2585	5.611	5.591	5.637	5.586	5.642
-2575	5.535	5.529	5.542	5.527	5.544
-2565	5.488	5.484	5.491	5.484	5.492
-2555	5.514	5.512	5.517	5.512	5.518
-2545	5.439	5.423	5.451	5.420	5.454
-2535	5.533	5.527	5.540	5.526	5.541
-2525	5.636	5.611	5.669	5.605	5.675
-2515	5.659	5.630	5.698	5.623	5.705
-2505	5.629	5.606	5.661	5.600	5.666
-2495	5.802	5.746	5.877	5.733	5.890
-2485	5.990	5.899	6.111	5.878	6.132
-2475	5.824	5.764	5.904	5.750	5.918
-2465	5.844	5.781	5.929	5.766	5.944
-2455	5.873	5.804	5.965	5.788	5.982
-2445	5.853	5.787	5.940	5.772	5.955
-2435	5.494	5.492	5.496	5.492	5.497
-2425	5.549	5.540	5.560	5.538	5.562
-2415	5.369	5.336	5.394	5.330	5.400
-2405	5.595	5.578	5.618	5.574	5.622
-2395	5.340	5.299	5.370	5.292	5.378
-2385	5.647	5.620	5.683	5.614	5.689
-2375	5.271	5.213	5.315	5.203	5.325
-2365	5.285	5.230	5.326	5.221	5.335
-2355	5.681	5.648	5.726	5.640	5.734
-2345	5.436	5.419	5.448	5.416	5.451
-2335	5.375	5.343	5.399	5.338	5.405
-2325	5.270	5.211	5.313	5.201	5.324
-2315	5.477	5.470	5.482	5.469	5.483
-2305	5.694	5.659	5.742	5.650	5.750
-2295	5.785	5.733	5.856	5.720	5.868
-2285	5.422	5.402	5.437	5.398	5.441
-2275	5.608	5.589	5.634	5.584	5.639
-2265	5.575	5.561	5.593	5.558	5.596
-2255	5.508	5.507	5.509	5.507	5.509
-2245	5.707	5.669	5.757	5.660	5.766
-2235	5.628	5.605	5.659	5.599	5.664
-2225	5.772	5.722	5.838	5.710	5.850
-2215	5.663	5.633	5.702	5.626	5.709
-2205	5.318	5.271	5.353	5.263	5.361
-2195	5.587	5.572	5.608	5.568	5.612
-2185	5.946	5.863	6.056	5.844	6.075

-2175	6.157	6.035	6.320	6.007	6.349
-2165	6.009	5.914	6.135	5.892	6.157
-2155	6.246	6.108	6.432	6.075	6.464
-2145	5.938	5.857	6.047	5.838	6.066
-2135	5.873	5.804	5.965	5.788	5.981
-2125	5.991	5.900	6.113	5.879	6.134
-2115	5.967	5.880	6.082	5.860	6.103
-2105	5.896	5.823	5.994	5.806	6.012
-2095	5.442	5.427	5.454	5.424	5.456
-2085	5.410	5.386	5.427	5.382	5.431
-2075	5.472	5.464	5.478	5.462	5.479
-2065	5.838	5.775	5.921	5.761	5.936
-2055	5.717	5.677	5.770	5.668	5.780
-2045	5.706	5.668	5.756	5.659	5.765
-2035	5.602	5.584	5.626	5.579	5.631
-2025	6.201	6.070	6.375	6.040	6.405
-2015	5.795	5.740	5.867	5.727	5.880
-2005	5.886	5.815	5.982	5.798	5.999
-1995	5.886	5.814	5.981	5.798	5.998
-1985	5.622	5.600	5.651	5.595	5.657
-1975	5.674	5.642	5.716	5.635	5.724
-1965	5.804	5.748	5.879	5.735	5.892
-1955	5.408	5.384	5.426	5.380	5.430
-1945	5.569	5.556	5.585	5.554	5.588
-1935	5.716	5.676	5.769	5.667	5.778
-1925	5.475	5.467	5.480	5.466	5.481
-1915	5.450	5.437	5.460	5.434	5.462
-1905	5.550	5.541	5.561	5.539	5.563
-1895	5.283	5.228	5.324	5.218	5.334
-1885	5.651	5.623	5.688	5.617	5.694
-1875	5.275	5.218	5.318	5.208	5.328
-1865	5.355	5.318	5.383	5.311	5.389
-1855	5.280	5.225	5.322	5.215	5.332
-1845	5.545	5.537	5.555	5.535	5.557
-1835	5.626	5.603	5.656	5.598	5.662
-1825	5.479	5.472	5.483	5.471	5.484
-1815	5.538	5.532	5.546	5.530	5.548
-1805	5.594	5.577	5.617	5.573	5.621
-1795	5.512	5.510	5.514	5.510	5.514
-1785	5.566	5.554	5.582	5.552	5.584
-1775	5.394	5.367	5.415	5.362	5.420
-1765	5.328	5.284	5.361	5.277	5.369

-1755	5.789	5.735	5.860	5.723	5.872
-1745	5.741	5.697	5.800	5.686	5.811
-1735	5.602	5.584	5.626	5.579	5.631
-1725	5.676	5.644	5.719	5.637	5.727
-1715	5.455	5.443	5.464	5.441	5.466
-1705	5.610	5.590	5.637	5.586	5.641
-1695	5.539	5.532	5.548	5.531	5.549
-1685	5.617	5.595	5.645	5.591	5.650
-1675	5.516	5.514	5.519	5.513	5.519
-1665	5.487	5.482	5.490	5.482	5.491
-1655	5.436	5.420	5.449	5.417	5.452
-1645	5.628	5.605	5.659	5.599	5.664
-1635	5.722	5.681	5.776	5.672	5.786
-1625	5.513	5.511	5.515	5.511	5.516
-1615	5.622	5.600	5.652	5.595	5.657
-1605	5.380	5.349	5.403	5.344	5.408
-1595	5.258	5.197	5.304	5.186	5.315
-1585	5.445	5.431	5.456	5.428	5.459
-1575	5.200	5.124	5.257	5.111	5.270
-1565	5.319	5.274	5.354	5.265	5.362
-1555	5.245	5.181	5.293	5.169	5.305
-1545	5.213	5.140	5.267	5.127	5.280
-1535	5.359	5.323	5.386	5.317	5.393
-1525	5.369	5.336	5.394	5.330	5.400
-1515	5.307	5.258	5.344	5.249	5.352
-1505	5.481	5.476	5.486	5.475	5.487
-1495	5.431	5.413	5.445	5.410	5.448
-1485	5.348	5.310	5.377	5.303	5.384
-1475	5.703	5.666	5.753	5.657	5.762
-1465	5.413	5.390	5.430	5.386	5.434
-1455	5.400	5.374	5.419	5.369	5.424
-1445	5.447	5.433	5.458	5.431	5.460
-1435	5.694	5.659	5.742	5.650	5.750
-1425	5.574	5.561	5.591	5.558	5.594
-1415	5.596	5.579	5.620	5.575	5.624
-1405	5.580	5.566	5.599	5.562	5.602
-1395	5.484	5.479	5.488	5.478	5.489
-1385	5.902	5.827	6.001	5.810	6.018
-1375	5.694	5.659	5.742	5.650	5.750
-1365	5.663	5.634	5.703	5.627	5.710
-1355	5.700	5.663	5.749	5.655	5.757
-1345	5.616	5.595	5.644	5.590	5.649

-1335	5.278	5.222	5.320	5.212	5.330
-1325	5.152	5.065	5.218	5.049	5.233
-1315	5.284	5.229	5.325	5.220	5.335
-1305	5.269	5.211	5.313	5.200	5.323
-1295	5.362	5.326	5.388	5.320	5.394
-1285	5.134	5.041	5.203	5.025	5.219
-1275	5.506	5.506	5.506	5.505	5.507
-1265	5.460	5.449	5.468	5.447	5.470
-1255	5.383	5.352	5.405	5.347	5.411
-1245	5.348	5.310	5.377	5.303	5.384
-1235	5.384	5.355	5.407	5.349	5.412
-1225	5.477	5.470	5.482	5.469	5.483
-1215	5.443	5.427	5.454	5.425	5.457
-1205	5.379	5.347	5.402	5.342	5.407
-1195	5.312	5.264	5.348	5.255	5.356
-1185	5.196	5.120	5.254	5.106	5.267
-1175	5.184	5.105	5.244	5.091	5.258
-1165	5.054	4.941	5.138	4.922	5.157
-1155	5.333	5.291	5.365	5.283	5.372
-1145	5.590	5.574	5.612	5.570	5.616
-1135	5.342	5.301	5.372	5.294	5.379
-1125	5.417	5.395	5.433	5.391	5.437
-1115	5.454	5.441	5.463	5.439	5.465
-1105	5.369	5.335	5.394	5.329	5.400
-1095	5.335	5.293	5.367	5.285	5.374
-1085	5.168	5.084	5.230	5.069	5.245
-1075	5.431	5.413	5.445	5.410	5.448
-1065	5.325	5.280	5.358	5.272	5.366
-1055	5.296	5.244	5.335	5.235	5.344
-1045	5.089	4.985	5.166	4.967	5.185
-1035	5.309	5.260	5.345	5.252	5.354
-1025	5.356	5.319	5.384	5.313	5.390
-1015	5.104	5.004	5.179	4.987	5.196
-1005	5.025	4.906	5.115	4.885	5.136
-995	5.040	4.924	5.127	4.904	5.147
-985	5.202	5.126	5.258	5.113	5.271
-975	5.216	5.144	5.270	5.132	5.282
-965	5.033	4.916	5.121	4.895	5.142
-955	5.338	5.297	5.369	5.290	5.376
-945	5.005	4.880	5.098	4.858	5.120
-935	5.119	5.023	5.191	5.006	5.208
-925	5.251	5.187	5.298	5.176	5.309

-915	5.137	5.046	5.206	5.030	5.222
-905	4.987	4.859	5.084	4.836	5.106
-895	5.155	5.068	5.220	5.053	5.236
-885	5.068	4.959	5.149	4.940	5.168
-875	5.352	5.315	5.381	5.308	5.387
-865	5.167	5.083	5.230	5.069	5.245
-855	5.291	5.239	5.331	5.229	5.340
-845	5.534	5.528	5.541	5.527	5.543
-835	5.722	5.681	5.777	5.672	5.786
-825	5.385	5.356	5.407	5.350	5.412
-815	5.533	5.527	5.540	5.526	5.541
-805	5.252	5.189	5.299	5.178	5.310
-795	5.460	5.449	5.468	5.447	5.470
-785	5.511	5.510	5.513	5.509	5.513
-775	5.487	5.483	5.490	5.482	5.491
-765	5.483	5.478	5.487	5.477	5.488
-755	5.450	5.437	5.460	5.434	5.462
-745	5.301	5.251	5.339	5.242	5.348
-735	5.429	5.410	5.443	5.407	5.446
-725	5.576	5.563	5.595	5.560	5.598
-715	5.586	5.570	5.606	5.567	5.610
-705	5.315	5.267	5.350	5.259	5.358
-695	5.320	5.274	5.354	5.266	5.362
-685	5.278	5.221	5.320	5.211	5.330
-675	5.276	5.219	5.319	5.209	5.329
-665	5.542	5.535	5.552	5.533	5.554
-655	5.702	5.665	5.751	5.656	5.760
-645	5.418	5.397	5.434	5.393	5.438
-635	5.834	5.772	5.917	5.758	5.931
-625	5.346	5.307	5.376	5.300	5.383
-615	5.650	5.623	5.687	5.616	5.693
-605	5.598	5.581	5.622	5.577	5.626
-595	5.389	5.361	5.411	5.356	5.416
-585	5.394	5.366	5.414	5.361	5.419
-575	5.450	5.437	5.460	5.434	5.462
-565	5.549	5.540	5.560	5.538	5.562
-555	5.213	5.141	5.267	5.128	5.280
-545	5.443	5.428	5.454	5.425	5.457
-535	5.583	5.568	5.603	5.565	5.606
-525	5.529	5.524	5.535	5.523	5.536
-515	5.437	5.421	5.450	5.418	5.453
-505	5.676	5.644	5.719	5.636	5.727

-495	5.450	5.437	5.460	5.434	5.462
-485	5.408	5.384	5.426	5.380	5.430
-475	5.585	5.570	5.605	5.566	5.609
-465	5.561	5.551	5.576	5.548	5.578
-455	5.195	5.118	5.253	5.104	5.266
-445	5.531	5.526	5.538	5.525	5.539
-435	5.296	5.244	5.335	5.235	5.344
-425	5.494	5.491	5.495	5.491	5.496
-415	5.625	5.602	5.655	5.597	5.660
-405	5.602	5.584	5.627	5.579	5.631
-395	5.619	5.597	5.647	5.592	5.652
-385	5.518	5.515	5.522	5.515	5.522
-375	5.933	5.853	6.040	5.834	6.059
-365	5.865	5.797	5.955	5.781	5.971
-355	5.713	5.674	5.766	5.665	5.775
-345	5.727	5.685	5.783	5.675	5.792
-335	5.575	5.562	5.593	5.559	5.596
-325	5.766	5.717	5.832	5.706	5.843
-315	5.662	5.632	5.701	5.625	5.708
-305	5.277	5.220	5.319	5.210	5.329
-295	5.506	5.506	5.507	5.506	5.507
-285	5.577	5.563	5.595	5.560	5.599
-275	5.583	5.568	5.602	5.564	5.606
-265	5.225	5.155	5.277	5.143	5.289
-255	5.384	5.354	5.406	5.349	5.412
-245	5.109	5.010	5.183	4.993	5.200
-235	5.273	5.215	5.316	5.205	5.326
-225	5.428	5.410	5.442	5.406	5.446
-215	5.316	5.269	5.351	5.260	5.359
-205	5.431	5.413	5.444	5.409	5.448
-195	5.575	5.562	5.593	5.559	5.597
-185	5.556	5.546	5.569	5.544	5.572
-175	5.648	5.621	5.683	5.614	5.690
-165	5.840	5.777	5.924	5.763	5.939
-155	5.772	5.722	5.839	5.710	5.851
-145	5.485	5.480	5.489	5.480	5.489
-135	5.502	5.501	5.502	5.501	5.502
-125	5.682	5.648	5.726	5.641	5.734
-115	5.497	5.495	5.498	5.495	5.498
-105	5.745	5.700	5.805	5.689	5.815
-95	6.059	5.955	6.197	5.931	6.222
-85	5.745	5.700	5.806	5.690	5.816

-75	5.785	5.733	5.855	5.720	5.868
-65	5.639	5.614	5.673	5.608	5.679
-55	5.796	5.741	5.869	5.729	5.882
-45	5.675	5.643	5.718	5.636	5.726
-35	5.789	5.735	5.860	5.723	5.872
-25	5.824	5.764	5.904	5.750	5.918
-15	5.832	5.771	5.914	5.757	5.929
-5	5.625	5.602	5.655	5.597	5.660
5	5.749	5.703	5.810	5.693	5.821
15	6.121	6.006	6.275	5.979	6.303
25	5.732	5.690	5.789	5.680	5.799
35	5.504	5.504	5.504	5.504	5.504
45	5.489	5.485	5.491	5.484	5.492
55	5.878	5.808	5.972	5.792	5.988
65	5.520	5.517	5.524	5.516	5.525
75	5.490	5.486	5.492	5.486	5.493
85	5.433	5.415	5.446	5.412	5.449
95	5.362	5.327	5.388	5.320	5.395
105	5.491	5.487	5.493	5.487	5.494
115	5.519	5.516	5.523	5.515	5.523
125	5.654	5.626	5.691	5.619	5.698
135	5.371	5.338	5.396	5.333	5.402
145	5.937	5.856	6.045	5.837	6.064
155	5.593	5.576	5.615	5.572	5.619
165	5.796	5.741	5.869	5.728	5.881
175	5.540	5.533	5.549	5.532	5.551
185	5.841	5.778	5.925	5.763	5.940
195	5.901	5.827	6.000	5.809	6.017
205	5.669	5.638	5.710	5.631	5.717
215	6.002	5.909	6.126	5.887	6.148
225	5.926	5.847	6.032	5.829	6.050
235	5.815	5.757	5.893	5.743	5.906
245	5.861	5.794	5.950	5.778	5.965
255	5.811	5.753	5.888	5.740	5.901
265	5.942	5.860	6.052	5.841	6.071
275	6.253	6.113	6.440	6.080	6.473
285	5.798	5.743	5.871	5.730	5.884
295	5.954	5.870	6.067	5.850	6.086
305	6.006	5.912	6.131	5.890	6.153
315	5.916	5.839	6.019	5.821	6.037
325	5.636	5.611	5.669	5.605	5.675
335	5.605	5.586	5.631	5.582	5.635

345	5.713	5.674	5.765	5.665	5.774
355	5.803	5.747	5.878	5.734	5.891
365	5.536	5.530	5.544	5.529	5.546
375	5.866	5.798	5.957	5.783	5.972
385	5.581	5.566	5.600	5.563	5.603
395	5.838	5.775	5.921	5.761	5.935
405	5.256	5.194	5.302	5.183	5.313
415	5.996	5.904	6.119	5.883	6.141
425	5.919	5.841	6.022	5.823	6.040
435	5.619	5.598	5.648	5.593	5.653
445	5.594	5.577	5.616	5.573	5.620
455	5.778	5.727	5.847	5.715	5.859
465	5.847	5.783	5.933	5.768	5.948
475	5.689	5.654	5.735	5.646	5.743
485	5.508	5.507	5.509	5.507	5.509
495	5.401	5.376	5.421	5.372	5.425
505	5.453	5.440	5.462	5.438	5.464
515	5.367	5.333	5.393	5.327	5.399
525	5.340	5.299	5.371	5.292	5.378
535	5.328	5.284	5.361	5.276	5.368
545	5.632	5.608	5.664	5.602	5.669
555	5.628	5.605	5.659	5.599	5.664
565	5.384	5.354	5.406	5.349	5.412
575	5.379	5.348	5.402	5.342	5.408
585	5.489	5.485	5.492	5.485	5.492
595	5.296	5.244	5.335	5.235	5.344
605	5.328	5.285	5.361	5.277	5.369
615	5.262	5.202	5.307	5.191	5.318
625	5.589	5.573	5.611	5.570	5.615
635	5.419	5.397	5.435	5.394	5.438
645	5.305	5.255	5.342	5.246	5.351
655	5.436	5.419	5.449	5.416	5.452
665	5.774	5.724	5.842	5.712	5.854
675	5.753	5.706	5.815	5.695	5.826
685	5.856	5.790	5.943	5.774	5.959
695	5.854	5.788	5.941	5.773	5.957
705	5.989	5.899	6.111	5.877	6.132
715	6.316	6.164	6.519	6.129	6.554
725	5.696	5.660	5.744	5.652	5.752
735	6.007	5.913	6.133	5.891	6.155
745	5.718	5.678	5.772	5.669	5.781
755	5.557	5.547	5.570	5.544	5.572

765	5.719	5.679	5.773	5.669	5.782
775	5.461	5.451	5.469	5.449	5.471
785	5.746	5.701	5.806	5.690	5.817
795	5.810	5.753	5.887	5.739	5.900
805	5.753	5.706	5.815	5.695	5.826
815	5.593	5.577	5.616	5.573	5.620
825	5.566	5.554	5.581	5.551	5.584
835	5.647	5.621	5.683	5.614	5.690
845	5.738	5.695	5.797	5.684	5.807
855	5.937	5.856	6.045	5.837	6.064
865	5.617	5.596	5.645	5.591	5.650
875	5.797	5.742	5.870	5.729	5.882
885	5.362	5.326	5.388	5.320	5.395
895	5.496	5.494	5.497	5.493	5.498
905	5.793	5.739	5.865	5.726	5.877
915	5.899	5.825	5.997	5.808	6.014
925	5.416	5.394	5.432	5.390	5.436
935	5.661	5.632	5.701	5.625	5.708
945	5.607	5.588	5.633	5.583	5.637
955	5.889	5.817	5.985	5.800	6.001
965	5.754	5.707	5.816	5.696	5.827
975	5.421	5.400	5.436	5.396	5.440
985	5.303	5.253	5.341	5.244	5.349
995	5.437	5.420	5.449	5.417	5.452
1005	5.097	4.996	5.173	4.978	5.191
1015	5.311	5.263	5.347	5.255	5.356
1025	5.286	5.232	5.327	5.222	5.336
1035	5.505	5.505	5.505	5.505	5.505
1045	5.615	5.594	5.642	5.589	5.647
1055	5.271	5.213	5.315	5.203	5.325
1065	5.568	5.556	5.583	5.553	5.586
1075	5.552	5.543	5.564	5.541	5.567
1085	5.650	5.622	5.686	5.616	5.692
1095	5.497	5.495	5.498	5.495	5.498
1105	5.640	5.614	5.674	5.608	5.680
1115	5.759	5.712	5.823	5.700	5.834
1125	5.530	5.525	5.537	5.524	5.538
1135	5.754	5.707	5.817	5.696	5.828
1145	5.733	5.690	5.790	5.680	5.800
1155	5.578	5.564	5.596	5.561	5.600
1165	5.371	5.338	5.396	5.333	5.402
1175	5.587	5.571	5.608	5.568	5.611

1185	5.994	5.902	6.116	5.881	6.137
1195	5.689	5.654	5.735	5.646	5.743
1205	5.869	5.800	5.960	5.785	5.976
1215	5.573	5.560	5.591	5.557	5.594
1225	5.724	5.683	5.779	5.674	5.789
1235	5.450	5.436	5.460	5.434	5.462
1245	5.676	5.644	5.719	5.636	5.726
1255	5.582	5.567	5.601	5.564	5.605
1265	5.389	5.361	5.411	5.356	5.416
1275	5.554	5.545	5.567	5.543	5.569
1285	5.583	5.568	5.603	5.565	5.607
1295	5.629	5.605	5.660	5.600	5.665
1305	5.811	5.754	5.888	5.740	5.901
1315	5.802	5.746	5.876	5.733	5.889
1325	5.879	5.809	5.972	5.792	5.989
1335	5.531	5.526	5.538	5.525	5.540
1345	5.650	5.622	5.686	5.616	5.693
1355	5.488	5.485	5.491	5.484	5.492
1365	5.480	5.473	5.484	5.472	5.485
1375	5.434	5.416	5.447	5.413	5.450
1385	5.471	5.463	5.477	5.461	5.478
1395	5.694	5.658	5.741	5.650	5.750
1405	5.638	5.613	5.671	5.607	5.677
1415	5.361	5.326	5.388	5.319	5.394
1425	5.431	5.412	5.444	5.409	5.448
1435	5.290	5.236	5.330	5.227	5.339
1445	5.365	5.330	5.391	5.324	5.397
1455	5.268	5.209	5.312	5.198	5.322
1465	5.607	5.588	5.633	5.584	5.638
1475	5.633	5.608	5.665	5.603	5.670
1485	5.393	5.366	5.414	5.361	5.419
1495	5.513	5.511	5.515	5.511	5.515
1505	5.408	5.385	5.426	5.380	5.430
1515	5.649	5.622	5.685	5.615	5.691
1525	5.574	5.561	5.592	5.558	5.595
1535	5.928	5.849	6.034	5.830	6.052
1545	5.795	5.740	5.867	5.728	5.880
1555	5.743	5.698	5.803	5.688	5.813
1565	5.793	5.739	5.865	5.726	5.878
1575	5.733	5.690	5.790	5.680	5.800
1585	5.507	5.506	5.507	5.506	5.507
1595	5.492	5.489	5.494	5.488	5.495

1605	5.577	5.563	5.596	5.560	5.599
1615	5.691	5.656	5.738	5.648	5.746
1625	5.404	5.379	5.422	5.374	5.427
1635	5.548	5.540	5.559	5.538	5.561
1645	5.450	5.436	5.460	5.434	5.462
1655	5.527	5.523	5.533	5.522	5.534
1665	5.414	5.391	5.430	5.387	5.434
1675	5.574	5.561	5.591	5.557	5.594
1685	5.469	5.460	5.476	5.459	5.477
1695	5.307	5.258	5.344	5.249	5.352
1705	5.547	5.539	5.558	5.537	5.560
1715	5.486	5.481	5.489	5.480	5.490
1725	5.671	5.640	5.713	5.632	5.720
1735	5.315	5.268	5.350	5.260	5.358
1745	5.353	5.315	5.381	5.308	5.387
1755	5.184	5.105	5.244	5.091	5.258
1765	5.172	5.090	5.234	5.075	5.249
1775	5.175	5.093	5.237	5.079	5.251
1785	5.222	5.151	5.274	5.139	5.287
1795	5.332	5.290	5.364	5.282	5.372
1805	5.189	5.111	5.248	5.097	5.262
1815	5.066	4.957	5.148	4.938	5.167
1825	5.114	5.017	5.187	5.000	5.204
1835	5.283	5.228	5.324	5.219	5.334
1845	5.174	5.092	5.236	5.077	5.250
1855	5.257	5.196	5.303	5.185	5.314
1865	5.272	5.214	5.315	5.203	5.325
1875	5.318	5.272	5.353	5.264	5.361
1885	5.242	5.177	5.291	5.165	5.302
1895	5.380	5.350	5.403	5.344	5.409
1905	5.551	5.542	5.562	5.540	5.564
1915	5.428	5.409	5.442	5.405	5.445
1925	5.240	5.175	5.290	5.163	5.301
1935	5.285	5.231	5.326	5.221	5.336
1945	5.278	5.222	5.320	5.212	5.330
1955	5.354	5.317	5.382	5.310	5.389
1965	5.579	5.565	5.597	5.561	5.601
1975	5.486	5.482	5.490	5.481	5.490
1985	5.757	5.710	5.820	5.699	5.831
1995	5.819	5.760	5.898	5.747	5.912
2005	5.887	5.815	5.982	5.799	5.999

References

Barnston, A.G., Livezey, R.E., 1987. Classification, seasonality and persistence of low-frequency atmospheric circulation patterns. Mon. Weather Rev. 115, 1083−1126.

Bond, G., Kromer, B., Beer, J., Muscheler, R., Evans, M., Showers, W., Hoffmann, S., Lotti-Bond, R., Hajdas, I., Bonani, G., 2001. Persistent solar influence on North Atlantic climate during the Holocene. Science 294, 2130−2136.

Bond, G., Showers, W., Cheseby, M., Lotti, R., Almasi, P., deMenocal, P., Priore, P., Cullen, H., Hajdas, I., Bonani, G., 1997. A pervasive millennial- scale cycle in North Atlantic Holocene and Glacial climates. Science 278, 1257−1266.

Bond, G.C., Showers, W., Elliot, M., Evans, M., Lotti, R., Hajdas, I., Bonani, G., Johnson, S., 1999. The North Atlantic's 1-2 kyr climate rhythm: relation to Heinrich events, Dansgaard/Oeschger cycles and the Little Ice Age. Geophysical Monographs 112, 35−58.

Büntgen, U., Myglan, V.S., Ljungqvist, F.C., McCormick, M., di Cosmo, N., Sigl, M., Jungclaus, J., Wagner, S., Krusic, P.J., Esper, J., Kaplan, J.O., de Vaan, M.A.C., Luterbacher, J., Wacker, L., Tegel, W., Kirdyanov, A.V., 2016. Cooling and societal change during the Late Antique Little Ice Age from 536 to around 660 AD. Nature Geosci. 9, 231–236.

Churakova (Sidorova), O.V., Bryukhanova, M.V., Saurer, M., Boettger, T., Naurzbaev, M.M., Myglan, V.S., Vaganov, E.A., Hughes, M.K., Siegwolf, R.T.W., 2014. A cluster of stratospheric volcanic eruptions in the AD 530s recorded in Siberian tree rings. Global Planet. Change 122, 140−150.

Compo, G.P., Whitaker, J.S., Sardeshmukh, P.D., Matsui, N., Allan, R.J., Yin, X., Gleason, B.E., Vose, R.S., Rutledge, G., Bessemoulin, P., Brönnimann, S., Brunet, M., Crouthamel, R.I., Grant, A.N., Groisman, P.Y., Jones, P.D., Kruk, M.C., Kruger, A.C., Marshall, G.J., Maugeri, M., Mok, H.Y., Nordli, Ø., Ross, T.F., Trigo, R.M., Wang, X.L., Woodruff, S.D., Worley, S.J., 2011. The Twentieth Century Reanalysis Project. Q. J. R. Meteorol. Soc. 137, 1−28.

D'Arrigo, R., Frank, D., Jacoby, G., Pederson, N., 2001. Spatial response to major volcanic events in or about AD 536, 934 and 1258: frost rings and other dendrochronological evidence from Mongolia and northern siberia. Climatic Change 49, 239−246.

Enfield, D.B., Mestas-Nunez, A.M., Trimble, P.J., 2001. The Atlantic Multidecadal Oscillation and its relationship to rainfall and river flows in the continental U.S. Geophys. Res. Lett. 28, 2077−2080.

Eronen, M., Hyvärinen, H., Zetterberg, P., 1999. Holocene humidity changes in northern Finnish Lapland inferred from lake sediments and submerged Scots pines dated by tree rings. Holocene 9, 569–580.

Eronen, M., Zetterberg, P., Briffa, K.R., Lindholm, M., Meriläiinen, J., Timonen, M., 2002. The supra-long Scots pine tree-ring record for Finnish Lapland: Part 1, chronology construction and initial inferences. Holocene 12, 673–680.

Esper, J., Frank, D.C., Timonen, M., Zorita, E., Wilson, R.J.S., Luterbacher, J., Holzkämper, S., Fischer, N., Wagner, S., Nievergelt, D., Verstege, A., Büntgen, U., 2012. Orbital forcing of treering data. Nature Clim. Change 2, 862–866.

Grudd, H., 2008. Torneträsk tree-ring width and density AD 500–2004: a test of climatic sensitivity and a new 1500-year reconstruction of north Fennoscandian summers. Clim. Dyn. 31, 843–857.

Harris, I., Jones, P.D., Osborn, T.J., Lister, D.H., 2014. Updated high-resolution grids of monthly climatic observations–the CRU TS3. 10 Dataset. Int. J. Climatol. 34, 623−642.

Helama, S., Arppe, L., Timonen, M., Mielikäinen, K., Oinonen, M., 2018a. A 7.5 ka chronology of stable carbon isotopes from tree rings with implications for their use in palaeo-cloud reconstruction. Global Planet. Change 170, 20−33.

Helama, S., Arppe, L., Uusitalo, J., Holopainen, J., Mäkelä, H.M., Mäkinen, H., Mielikäinen, K., Nöjd P., Sutinen, R., Taavitsainen, J.-P., Timonen, M., Oinonen, M. 2018b. Volcanic dust veils from sixth century tree-ring isotopes linked to reduced irradiance, primary production and human health. Sci. Rep. 8, 1339. https://doi.org/10.1038/s41598-018-19760-w.

Helama, S., Jones, P.D., Briffa, K.R., 2017a. Dark Ages Cold Period: a literature review and directions for future research. Holocene 27, 1600−1606.

Helama, S., Jones, P.D., Briffa, K.R., 2017b. Limited Late Antique cooling. Nature Geosci. 10, 242−243.

Helama, S., Mielikäinen, K., Timonen, M., Eronen, M., 2008. Finnish supra-long tree-ring chronology extended to 5634 BC. Norwegian J. Geogr. 62, 271–277.

Helama, S., Macias Fauria, M., Mielikäinen, K., Timonen, M., Eronen M., 2010. Sub-Milankovitch solar forcing of past climates: mid and late Holocene perspectives. GSA Bull. 122, 1981−1988.

Helama, S., Saranpää, P., Pearson, C.L., Arppe, L., Holopainen, J., Mäkinen, H., Mielikäinen, K., Nöjd, P., Sutinen, R., Taavitsainen, J.-P., Timonen, M., Uusitalo, J., Oinonen, M., 2019. Frost rings in 1627 BC and AD 536 in subfossil pinewood from Finnish Lapland. Quaternary Sci. Rev. 204, 208−215.

Helama, S., Timonen, M., Lindholm, M. Meriläinen, J., Eronen, M., 2005. Extracting long-period climate fluctuations from tree-ring chronologies over timescales of centuries to millennia. Int. J. Climatol. 25, 1767−1779.

Hurrell, J.W., Deser, C., 2010. North Atlantic climate variability: the role of the North Atlantic Oscillation. J. Mar. Systems 78, 28-41.

Jones, P.D., Lister, D.H., Osborn, T.J., Harpham, C., Salmon, M., Morice, C.P., 2012. Hemispheric and large-scale land surface air temperature variations: an extensive revision and an update to 2010. J. Geophys. Res. 117, D05127. https://doi.10.1029/2011JD017139.

Kobashi, T., Menviel, L., Jeltsch-Thömmes, A., Vinther, B.M., Box, J.E., Muscheler, R., Nakaegawa, T., Pfister, P.L., Döring, M., Leuenberger, M., Wanner, H., Ohmura, A. 2017. Volcanic influence on

centennial to millennial Holocene Greenland temperature change. Sci. Rep. 7, 1441. DOI:10.1038/s41598-017-01451-7.

Macias-Fauria, M., Grinsted, A., Helama, S., Holopainen, J., 2012. Persistence matters: Estimation of the statistical significance of paleoclimatic reconstruction statistics from autocorrelated time series. Dendrochronologia 30, 179−187.

Matskovsky, V.V., Helama, S., 2014. Testing long-term summer temperature reconstruction based on maximum density chronologies obtained by reanalysis of tree-ring data sets from northernmost Sweden and Finland. Clim. Past 10, 1473−1487.

Mayewski, P.A., Rohling, E.E., Stager, C.J., Karlén, W., Maasch, K.A., Meeker, L.D., Meyerson, E.A., Gasse, F., van Kreveld, S., Holmgren, K., Lee-Thorp, J., Rosqvist, G., Rack, F., Staubwasser, M., Schneider, R.R., Steig, E.J., 2004. Holocene climate variability. Quaternary Res. 62, 243−255.

Melvin, T.M., Grudd, H., Briffa, K.R., 2013. Potential bias in “updating” tree-ring chronologies using regional curve standardisation: Re-processing 1500 years of Torneträsk density and ring-width data. Holocene 23, 364–373.

Plummer, C.T., Curran, M.A.J., van Ommen, T.D., Rasmussen, S.O., Moy, A.D., Vance, T.R., Clausen, H.B., Vinther, B.M., Mayewski, P.A., 2012. An independently dated 2000-yr volcanic record from Law Dome, East Antarctica, including a new perspective on the dating of the 1450s CE eruption of Kuwae, Vanuatu. Clim. Past 8, 1929−1940.

Salzer, M.W., Hughes, M.K., 2007. Bristlecone pine tree rings and volcanic eruptions over the last 5000 yr. Quaternary Res. 67, 57−68.

Schweingruber, F.H., Bartholin, T., Schär, E., Briffa, K.R., 1988. Radiodensitometric dendroclimatological conifer chronologies from Lapland (Scandinavia) and the Alps (Switzerland). Boreas 17, 559–566.

Sigl, M.., Winstrup, M., McConnell, J.R., Welten, K.C., Plunkett, G., Ludlow, F., Büntgen, U., Caffee, M., Chellman, N., Dahl-Jensen, D., Fischer, H., Kipfstuhl, S., Kostick, C., Maselli, O.J., Mekhaldi, F., Mulvaney, R., Muscheler, R., Pasteris, D.R., Pilcher, J.R., Salzer, M., Schüpbach, S., Steffensen, J.P., Vinther, B.M., Woodruff, T.E., 2015. Timing and climate forcing of volcanic eruptions for the past 2,500 years. Nature 523, 543−549.

Steinhilber, F., Beer, J., Fröhlich, C. 2009. Total solar irradiance during the Holocene. Geophys. Res. Lett. 36, L19704. DOI: 10.1029/2009GL040142.

Stoffel, M., Khodri, M.,Corona, C., Guillet, S., Poulain, V., Bekki, S., Guiot, J., Luckman, B.H., Oppenheimer, C., Lebas, N., Beniston, M., Masson-Delmotte, V., 2015. Estimates of volcanic-induced cooling in the Northern Hemisphere over the past 1,500 years. Nature Geosci. 8, 784−788.

Trenberth, K.E., Paolino, D.A., 1980. The Northern Hemisphere sea level pressure data set: Trends, errors, and discontinuities. Mon. Weather Rev. 108, 855−872.

Trouet, V., van Oldenborgh, G.J., 2013. KNMI Climate Explorer: a web-based research tool for high-resolution paleoclimatology. Tree-Ring Res. 69, 3−13.

Wanner, H., Solomina, O., Grosjean, M., Ritz, S.P., Jetel, M., 2011. Structure and origin of Holocene cold events. Quaternary Sci. Rev. 30, 3109–3123.
